# ﻿First record of the genus *Catatemnus* Beier, 1932 from China, with the description of six new species (Pseudoscorpiones, Atemnidae)

**DOI:** 10.3897/zookeys.1168.100798

**Published:** 2023-07-03

**Authors:** Yanmeng Hou, Lingchen Zhao, Feng Zhang

**Affiliations:** 1 The Key Laboratory of Zoological Systematics and Application, Institute of Life Science and Green Development, College of Life sciences, Hebei University, Baoding, Hebei 071002, China Hebei University Baoding China; 2 Changzhi Tunliu District No. 6 Middle School, Changzhi, Shanxi 046100, China Changzhi Tunliu District No. 6 Middle School Changzhi China

**Keywords:** morphology, new species, pseudoscorpion, taxonomy

## Abstract

The genus *Catatemnus* Beier, 1932 is reported for the first time from China and includes six new species: *C.huae***sp. nov.** from Hainan Island, *C.laminosus***sp. nov.**, *C.ramus***sp. nov.**, *C.scaber***sp. nov.**, and *C.tengchongensis***sp. nov.** all from Yunnan Province, and *C.tibetanus***sp. nov.** from Xizang Autonomous Region. Descriptions and illustrations of all the new species are provided.

## ﻿Introduction

The pseudoscorpion family Atemnidae Kishida, 1929 comprises two subfamilies, 20 extant genera and one fossil genus (*Progonatemnus* Beier, 1955; WPC 2023). To date, 188 species have been reported worldwide, of which ten species and four genera are known from China: *Anatemnuschaozhouensis* Hu & Zhang, 2012, *A.hemuensis* Li & Wang, 2021, *A.orites* (Thorell, 1889), and *A.reni* Gao & Zhang, 2016; *Anatemnushamiensis* Zhao, Gao & Zhang, 2020, *A.limuensis* Hu & Zhang, 2012, *A.niger* Zhao, Gao & Zhang, 2020, and *A.politus* (Simon, 1878); *Diplotemnusbalcanicus* (Redikorzev, 1928) and *Paratemnoidessinensis* (Beier, 1932) (Li and Wang 2021; WPC 2023).

*Catatemnus* Beier, 1932, one of the genera within the family Atemnidae, is currently represented by 13 species, which are widespread in Africa (Angola, Cameroon, Congo, Côte d’Ivoire, Guinea, Kenya, Mayotte, Nigeria, Somalia, South Africa, Tanzania, Uganda and Zimbabwe), south Asia (India), and southeast Asia (Indonesia and Myanmar); none are recorded in China (WPC 2023).

The genus *Catatemnus* can be diagnosed as follows: carapace longer than broad, with two eyespots or no eyes, surface smooth and with a median furrow; tergites XI incompletely divided in some species and other tergites undivided; galea present and with dentations or branchlets apically; rallum composed of four blades and with one or two dentated blades; pedipalp stout, surface smooth or with granulations on prolateral surface; palpal trochanter with two well-developed granular tubercles; trichobothrium it away from fixed chelal fingertip, but distance between it and fingertip shorter to distance between ist and isb; st closer to sb than to t generally; legs moderately stout, a long tactile setae of leg IV situated near the base of tarsal segment (Beier 1932; Murthy and Ananthakrishnan 1977).

During the identification of atemnid pseudoscorpion specimens collected from 2011 to 2018, six new species belonging to *Catatemnus* were found, which are presented with detailed diagnoses, descriptions, and illustrations.

## ﻿Materials and methods

The specimens are preserved in 75% ethanol and deposited in the Museum of Hebei University (**MHBU**) (Baoding, China) and the Museum of Southwest University (**MSWU**) (Chongqing, China). Photographs were taken with a Leica M205A stereomicroscope equipped with a Leica DFC550 camera and the LAS software v. 4.6, and the Leica M205A stereomicroscope with a drawing tube was used for drawings and measurements. The chela and the chelal hand were measured in ventral view. Detailed examination was carried out with an Olympus BX53 compound light microscope. Temporary slide mounts were made in glycerol.

Terminology and measurements mostly follow Chamberlin (1931), with some minor modifications to the terminology of trichobothria (Harvey 1992), chelicerae (Judson 2007), and male genitalia (Klausen 2005). All measurements are given in mm unless noted otherwise.

The following abbreviations are used for the trichobothria: **b** = basal; **sb** = sub-basal; **st** = sub-terminal; **t** = terminal; **ib** = interior basal; **isb** = interior sub-basal; **ist** = interior sub-terminal; **it** = interior terminal; **eb** = exterior basal; **esb** = exterior sub-basal; **est** = exterior sub-terminal; **et** = exterior terminal. Cheliceral setae: **es** = exterior seta; **is** = interior seta; **ls** = laminal seta; **bs** = basal seta. Male genitalia: **br** = hooked branch; **c** = sclerotized bar; **d** = longitudinal fold of medial diverticulum; **e** = ejaculatory canal atrium; **f** = lateral rods; **g** = dorsal apodeme; **h** = ventral diverticulum; **l** = lateral lip of lateral apodeme.

## ﻿Taxonomy

### ﻿Family Atemnidae Kishida, 1929

#### ﻿Genus Catatemnus

Taxon classificationAnimaliaPseudoscorpionesAtemnidae

Beier, 1932

5D3AD322-6285-5BE4-9D03-8F0E529D863D

##### Type species.

*Cheliferbirmanicus* Thorell, 1889, by original designation.

#### ﻿ Catatemnus huae sp. nov.

Taxon classificationAnimaliaPseudoscorpionesAtemnidae

ACB5C00C-722F-5B12-AED0-FAD4DA2DE82E

https://zoobank.org/3046D213-0100-4492-8883-CE96EE2393C9

**Figure 1.**
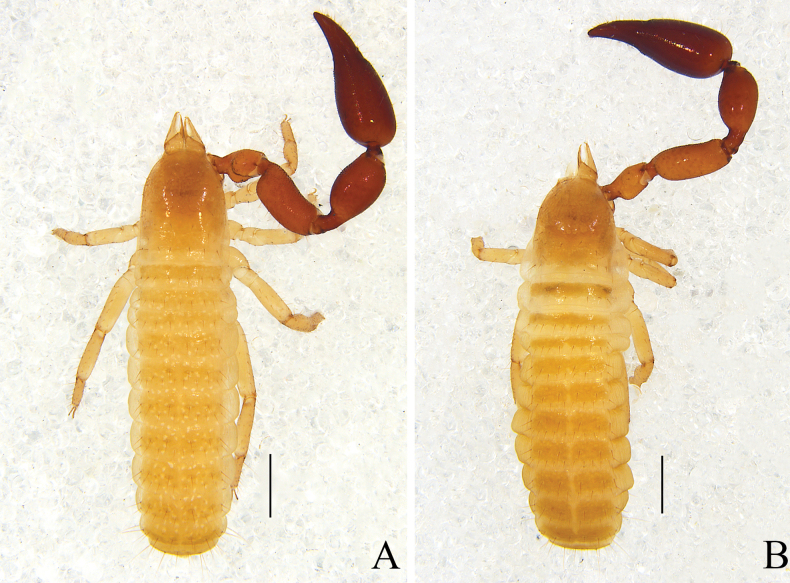
*Catatemnushuae* sp. nov. **A** holotype male, habitus (minus left pedipalp, left legs I and IV, dorsal view) **B** paratype female, habitus (minus left pedipalp, left legs I and III, right leg IV, dorsal view). Scale bars: 0.5 mm.

**Figure 2.**
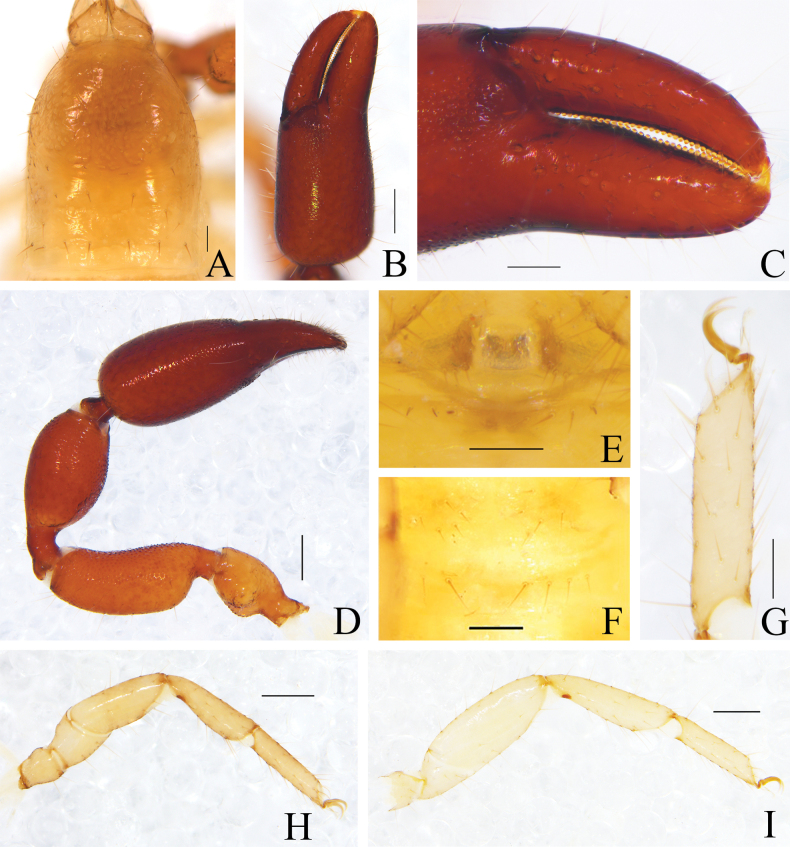
*Catatemnushuae* sp. nov., holotype male (**A–E, G–I**), paratype female (**F**) **A** carapace (dorsal view) **B** left chela (lateral view) **C** left chelal fingers (lateral view) **D** left pedipalp (dorsal view) **E** male genital area (ventral view) **F** female genital area (ventral view) **G** tarsus of left leg IV (lateral view) **H** left leg I (lateral view) **I** left leg IV (lateral view). Scale bars: 0.20 mm (**B, D, H, I**); 0.10 mm (**A, C, E–G**).

**Figure 3.**
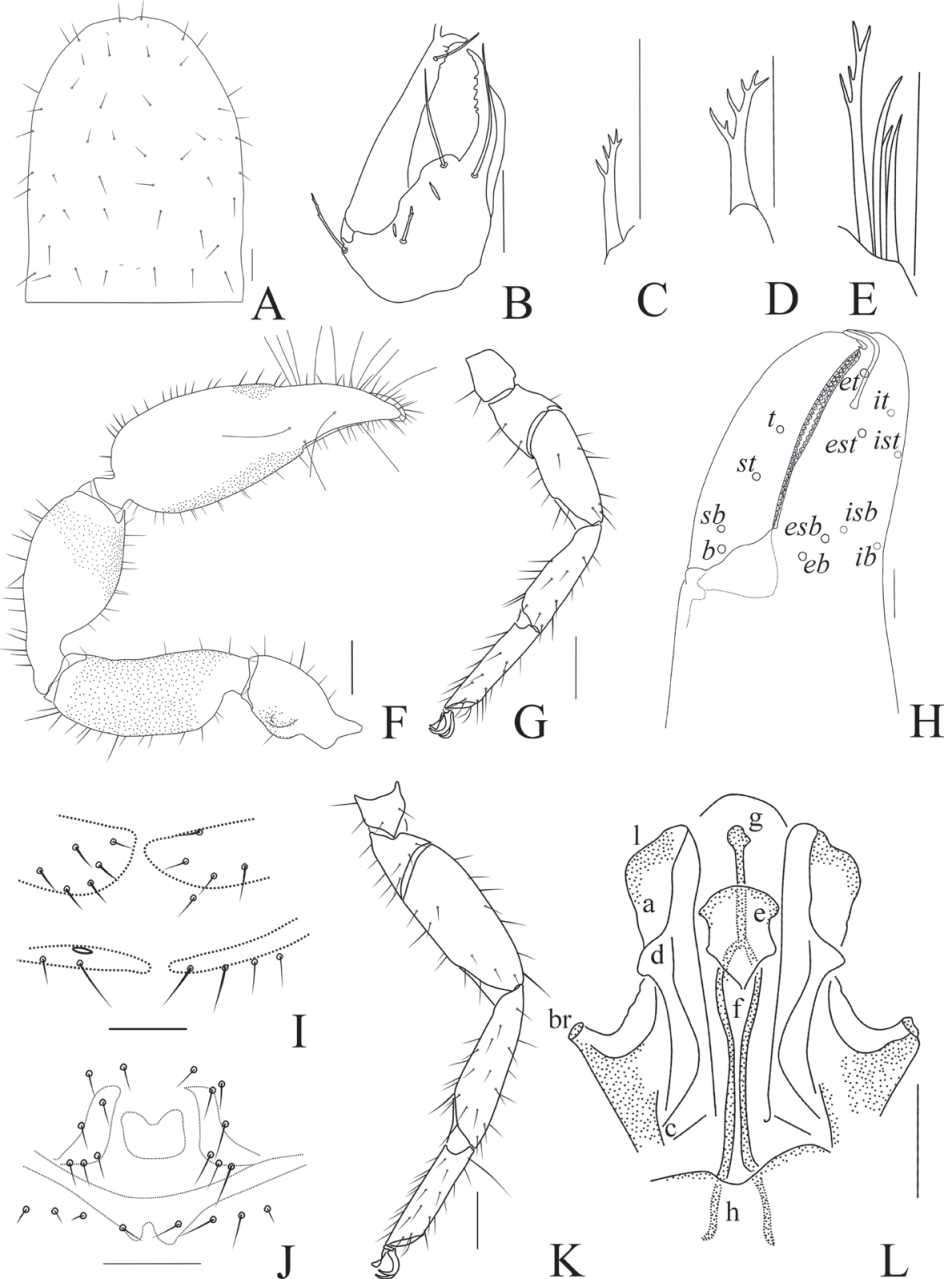
*Catatemnushuae* sp. nov., holotype male (**A–C, E–H, J–L**), paratype female (**D, I**) **A** carapace (dorsal view) **B** left chelicera (dorsal view) **C** male galea **D** female galea **E** rallum **F** left pedipalp (dorsal view) **G** left leg I (lateral view) **H** left chelal fingers (lateral view), with details of trichobothrial pattern **I** female genital area (ventral view) **J** male genital area (ventral view) **K** left leg IV (lateral view) **L** male genital organ. Scale bars: 0.20 mm (**F, G, K**); 0.10 mm (**A–E, H–J, L**). Abbreviations: **b** = basal; **sb** = sub-basal; **st** = sub-terminal; **t** = terminal; **ib** = interior basal; **isb** = interior sub-basal; **ist** = interior sub-terminal; **it** = interior terminal; **eb** = exterior basal; **esb** = exterior sub-basal; **est** = exterior sub-terminal; **et** = exterior terminal; **a** = lateral apodeme; **br** = hooked branch; **c** = sclerotized bar; **d** = longitudinal fold of medial diverticulum; **e** = ejaculatory canal atrium; **f** = lateral rods; **g** = dorsal apodeme; **h** = ventral diverticulum; **l** = lateral lip of lateral apodeme.

Figs 1 , 2 , 3 (胡氏猫伪蝎)

##### Type material.

***Holotype***: China • ♂; Hainan Province, Lingshui County, Xincun Town, Tonghai Village; 18°26.084'N, 110°0.495'E; 10 m a.s.l.; 19 May. 2011; Junfang Hu leg.; Ps.–MHBU–HA11051901. ***Paratype***: • 1♀; same data as the holotype; Ps.–MSWU–HA11051902.

##### Diagnosis (♂♀).

This new species is characterized by carapace smooth but with a faint medial furrow; with two faint eyespots situated near anterior margin of carapace. Palpal stout, femur (♂) 2.48×, (♀) 2.52×, chela with pedicel (♂) 3.05×, (♀) 2.93×, without pedicel (♂) 2.81×, (♀) 2.74× longer than broad; the retrolateral surface of trochanter, the prolateral surface of femur, patella and hand granular; male genitalia: the hooked branch (br) relatively small.

##### Description.

**Adult male** (Figs 1A, 2A–E, G–I, 3A–H, J–L). ***Color***: anterior half of carapace yellowish brown, paler in posterior half; pedipalps reddish brown, paler in female; tergites brown, paler in legs; remainder yellow. ***Carapace*** (Figs 2A, 3A): anterior half slightly darker than posterior half; surface smooth, but with a faint medial furrow; with two faint eyespots situated near anterior margin of carapace; anterior margin with six setae, posterior margin with seven setae, 47 in total, each seta acicular and very slightly curved; with a pair of lyrifissures on anterior margin and posterior margin, respectively. ***Chelicera*** (Fig. 3B, C, E): much smaller than carapace length; surface smooth; four setae (sbs absent; bs shorter than others) and two lyrifissures (exterior condylar lyrifissure and exterior lyrifissure) present on hand; movable finger with one galeal seta; bs and es dentate apically, is and ls long and acute. Fixed finger with six large retrorse teeth and three small apical teeth, movable finger with a long broadly rounded subapical lobe; galea present, slender, apically with five branchlets (Fig. 3C). Serrula interior connected to fixed finger for entire length, proximally modified to form velum, serrula exterior with 26 lamellae, the basal one longest; lamina exterior present. Rallum composed of four blades, the basal two blades shorter than others, the distal one dentated anteriorly, remainder smooth (Fig. 3E). ***Pedipalp*** (Figs 2B–D, 3F, H): stout, trochanter 1.85×, femur 2.48×, patella 2.14×, chela with pedicel (without pedicel) 3.05× (2.81×), hand with pedicel (without pedicel) 1.79× (1.55×) longer than broad; movable chelal finger 0.71× (0.82×) longer than hand with pedicel (without pedicel). Setae generally long and acuminate. Retrolateral surface of trochanter, the prolateral surface of femur, patella, and hand granular; trochanter with a rounded ventral tubercle and a well-developed conical dorsal tubercle. Fixed chelal finger with eight trichobothria, movable chelal finger with four trichobothria: eb and esb situated at base of fixed finger on retrolateral face, esb slightly distal to eb; ib and isb situated at base of fixed finger on prolateral face, isb slightly distal to ib; est in the middle of fixed finger; et near sub-distal of fixed finger; est closer to et than to esb; it slightly distal to est and proximal to et; ist situated proximal to est and distal to isb; it closer to ist than to fingertip; distance between est and esb farther to that of ist and isb, distance between it and fingertip nearly shorter to distance between ist and isb; b and sb situated at base of movable finger on retrolateral face; t in the middle of movable finger and at same level as est; sb closer to b than to st; st closer to t than to sb (Fig. 3H). Venom apparatus only present in fixed chelal finger, venom ducts curved and short, terminating in inflated nodus ramosus between et and est and closer to est. Both chelal fingers with a row of acute teeth, spaced contiguously along the margin, slightly rounded proximally: fixed chelal finger with 28 teeth; movable chelal finger with 35 teeth (nearly as large as teeth on fixed chelal finger); without accessory teeth (Fig. 3H). Sense spots absent; femur without long tactile setae. Movable chelal finger slightly curved in lateral view (Figs 2C, 3H). ***Opisthosoma***: generally typical, all setae long and acuminate, pleural membrane longitudinally striate, without setae. Tergites I and XI undivided and other tergites incompletely divided, tergal chaetotaxy I–XI: 9: 9: 4–4: 7–7: 9–8: 7–8: 7–8: 8–7: 8–6: 7–7+ (2T): 13 + (4T), arranged in two rows on tergites IV–XI. Sternites XI undivided and other sternites divided, sternal chaetotaxy IV–XI: 6–6: 8–7:8–8: 8–8: 6–7: 7–7: 7–7 (4T): 13 + (4T). Anus (tergite XII and sternite XII) without raised rim. Anterior genital operculum with seven setae on each side, posterior margin with four setae on each side and with one or two lyrifissures. Male genitalia (Figs 2E, 3J, L): the distal part (l) of lateral apodemes with a distinctive inner ridge curved into semicircle; the hooked branch (br) bowed distally and terminated in a plate-like tip; the proximal part with a distinct dark sclerotized bar (c), shorter and incurved; the longitudinal fold of medial diverticula (d) with a projection midway along its length, forming a lobe bulge; the ejaculatory canal atrium (e) not well-developed, curved distally; the lateral rods (f) long and diverging proximally; the tip of dorsal apodeme (g) completely joined; the ventral diverticulum (h) bilobed; genital atrium without genital setae. ***Legs*** (Figs 2G–I, 3G, K): typical, fairly smooth, slightly stout; junction between femora and patellae I and II oblique. Femoropatella of leg IV 3.13× longer than deep; tibia 3.93× longer than deep; with basal tactile setae on tarsal segment: tarsus 4.50× longer than deep (TS = 0.09–0.11); subterminal tarsal setae arcuate and acute. Arolium slightly shorter than claws, not divided; claws smooth.

**Adult Female** (Figs 1B, 2F, 3D, I): mostly same as the male, but a little larger in body size; anterior half of carapace yellowish brown, paler in posterior half; pedipalps reddish brown; tergites yellowish brown, paler in legs; remainder yellow. ***Carapace***: anterior margin with two setae, posterior margin with eight setae, 46 in total. ***Chelicera***: hand with four setae, bs and es dentate apically, is and ls long and acute; fixed finger with six large retrorse teeth and three small apical teeth, movable finger with two long broadly rounded subapical lobes; galea with six branchlets (Fig. 3D); serrula exterior with 21 lamellae; rallum with four blades. ***Pedipalp***: stout, the retrolateral surface of trochanter, the prolateral surface of femur, patella and base of chelal fingers granular; trochanter with two distinct conical dorsal tubercles; trochanter 1.88×, femur 2.52×, patella 2.20×, chela (with pedicel) 2.93×, chela (without pedicel) 2.74×, hand (without pedicel) 1.58× longer than broad, movable chelal finger 0.67× (0.75×) longer than hand with pedicel (without pedicel). ***Opisthosoma***: tergites I, II, and XI undivided, tergites III and IV incompletely divided and other tergites completely divided; tergal chaetotaxy (I–XI): 8: 11: 6–6: 8–7: 7–8: 9–8: 9–9: 8–8: 8–8: 14+ (2T): 10+ (4T); sternites XI undivided and other sternites divided, sternal chaetotaxy (IV–XI): 6–6: 8–8:8–9: 7–5: 8–6: 8–8: 8–9 (4T): 9 + (4T); genital structure (Figs 2F, 3I) simple, spermathecae provided with separated median cribriform plates.

**Dimensions** (length/breadth or, in the case of the legs, length/depth in mm). Male (female in parentheses): body length 3.19 (3.21). Carapace 0.87/0.66 (0.87/0.69). Pedipalp: trochanter 0.48/0.26 (0.49/0.26), femur 0.72/0.29 (0.78/0.31), patella 0.77/0.36 (0.77/0.35), chela (with pedicel) 1.28/0.42 (1.35/0.46), chela (without pedicel) 1.18 (1.26), hand (with pedicel) 0.75 (0.82), hand (without pedicel) 0.65 (0.73), movable finger length 0.53 (0.55). Leg I: trochanter 0.17/0.14 (0.16/0.15), femur 0.28/0.18 (0.27/0.19), patella 0.40/0.16 (0.40/0.16), tibia 0.40/0.12 (0.42/0.12), tarsus 0.38/0.08 (0.41/0.09). Leg IV: trochanter 0.30/0.16 (0.30/0.17), femoropatella 0.75/0.24 (0.79/0.25), tibia 0.59/0.15 (0.62/0.16), tarsus 0.45/0.10 (0.48/0.11).

##### Remarks.

Prior to this study, 13 species of *Catatemnus* have been recorded around the world, of which five come from Asia, but none have been recorded in China. The medial furrow on the carapace of *Catatemnushuae* sp. nov. was a little faint (but still visible) probably due to the pale body color.

*Catatemnushuae* sp. nov. can be distinguished from *C.birmanicus* (Thorell, 1889) by the stouter pedipalps (e.g., palpal femur (♀) 2.52× vs 2.36× longer than broad, length 0.78 mm vs 0.92 mm) and the traits of pedipalps (distinctly granular vs minutely granular); from *C.concavus* (With, 1906) by the stouter pedipalps (♀) (e.g., palpal femur 2.52× vs 2.29× longer than broad, length 0.78 mm vs 0.90 mm), the trait of galea (galea well-developed and with six branchlets vs galea undeveloped and partly lost) and the color of the tergites (pale yellow vs darker brown); from *C.monitor* (With, 1906) by stouter pedipalps (e.g., palpal femur (♂) 2.48× vs 2.25×, (♀) 2.52× vs 2.27× longer than broad, length (♂) 0.72 mm vs 1.01 mm, (♀) 0.78 mm vs 0.95 mm), the trait of eyespots (with two faint eyespots vs no eyespots visible) and the color of the dorsal surface of the body (pale yellow vs dark brown); from *C.nicobarensis* (With, 1906) by the stouter pedipalps (♂) (e.g., palpal femur 2.48× vs 2.14× longer than broad, length 0.72 mm vs 0.97 mm) and the traits of the palpal trochanter (anterior surface granular vs smooth or almost smooth); from *C.thorelli* (Balzan, 1892) by the trait of eyespots (with two faint eyespots vs with two distinct eyespots) and the traits of pedipalps (surface of all segments (except chelal fingers) granular vs surface of palps smooth) (Thorell 1889; Balzan 1891; With 1906; Beier 1932).

##### Distribution.

Known only from the type locality.

##### Etymology.

The new species is named after Junfang Hu, who collected the specimen for this study.

#### ﻿ Catatemnus laminosus sp. nov.

Taxon classificationAnimaliaPseudoscorpionesAtemnidae

A5D8C736-46BD-5CB9-8232-BFE1C4141A8C

https://zoobank.org/0B5CB61C-12FE-41D9-9CDA-BD4CAEF61237

**Figure 4.**
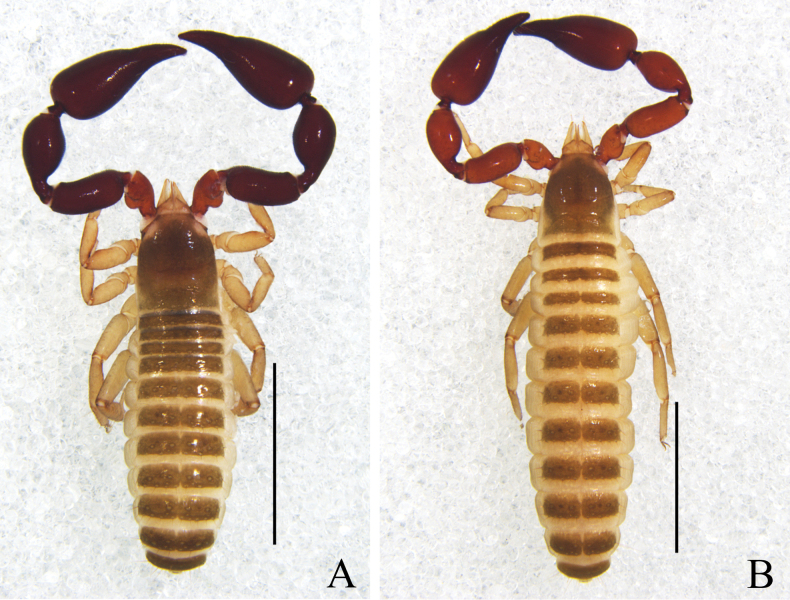
*Catatemnuslaminosus* sp. nov. **A** holotype male, habitus (dorsal view) **B** paratype female, habitus (dorsal view). Scale bars: 2.00 mm.

**Figure 5.**
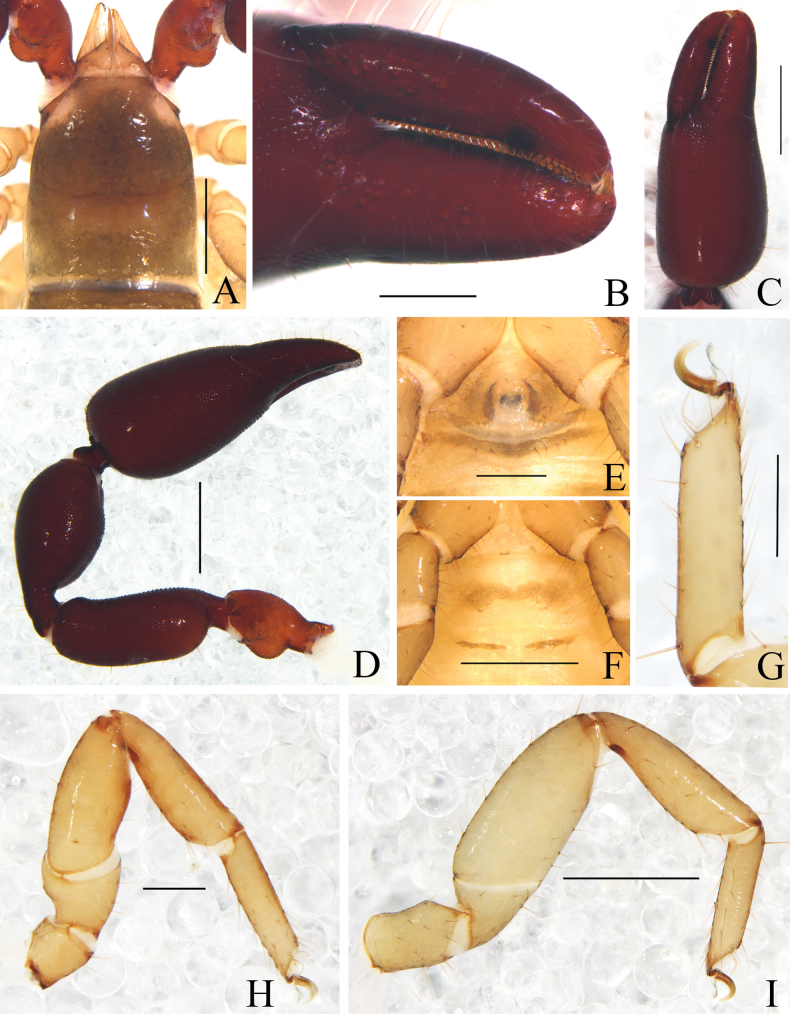
*Catatemnuslaminosus* sp. nov., holotype male (**A–E, G–I**), paratype female (**F**) **A** carapace (dorsal view) **B** left chela (lateral view) **C** left chelal fingers (lateral view) **D** left pedipalp (dorsal view) **E** male genital area (ventral view) **F** female genital area (ventral view) **G** tarsus of left leg IV (lateral view) **H** left leg I (lateral view) **I** left leg IV (lateral view). Scale bars: 0.50 mm (**A, C, D, F, I**); 0.20 mm (**B, E, G, H**).

**Figure 6.**
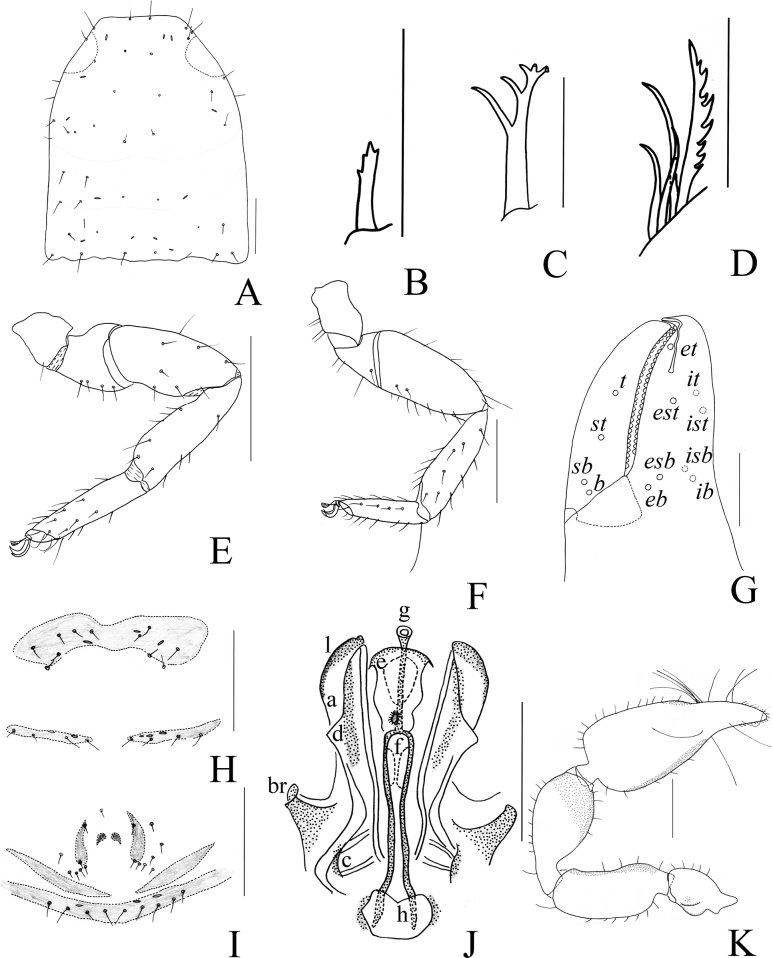
*Catatemnuslaminosus* sp. nov., holotype male (**A, B, D–G, I–K**), paratype female (**C, H**) **A** carapace (dorsal view) **B** male galea **C** female galea **D** rallum **E** left leg I (lateral view) **F** left leg IV (lateral view) **G** left chelal fingers (lateral view), with details of trichobothrial pattern **H** female genital area (ventral view) **I** male genital area (ventral view) **J** male genital organ **K** left pedipalp (dorsal view). Scale bars: 0.50 mm (**E, F, K**); 0.25 mm (**A, G–J**); 0.10 mm (**B–D**). Abbreviations: **b** = basal; **sb** = sub-basal; **st** = sub-terminal; **t** = terminal; **ib** = interior basal; **isb** = interior sub-basal; **ist** = interior sub-terminal; **it** = interior terminal; **eb** = exterior basal; **esb** = exterior sub-basal; **est** = exterior sub-terminal; **et** = exterior terminal; **a** = lateral apodeme; **br** = hooked branch; **c** = sclerotized bar; **d** = longitudinal fold of medial diverticulum; **e** = ejaculatory canal atrium; **f** = lateral rods; **g** = dorsal apodeme; **h** = ventral diverticulum; **l** = lateral lip of lateral apodeme.

Figs 4 , 5 , 6 (多层猫伪蝎)

##### Type material.

***Holotype***: China • ♂; Yunnan Province, Menghai County, Mengsong Township, Nabanhe River National Nature Reserve; 22°6.779'N, 100°35.109'E; 1813 m a.s.l.; 23 Jul. 2018; Chi Jin and Chen Zhang leg.; Ps.–MHBU–YNBN181701. ***Paratypes***: • 2♀; same data as the holotype; Ps.–MSWU–YNBN181702–181703.

##### Diagnosis (♂♀).

This new species is characterized by carapace smooth but with a medial furrow; with two distinct developed eyespots situated near anterior margin of carapace. Palpal femur (♂) 2.46×, (♀) 2.39–2.51×, chela with pedicel (♂) 2.68×, (♀) 2.54–2.57×, chela without pedicel (♂) 2.43×, (♀) 2.29–2.35× longer than broad; distance between est and esb farther or nearly equal to that of ist and isb; serrula exterior with 27 or 29 lamellae; male genitalia: the longitudinal fold of medial diverticula (d) with a projection midway along its length, forming a digitiform bulge.

##### Description.

**Adult male** (Figs 4A, 5A–E, G–I, 6A, B, D, E–G, I–K). ***Color***: carapace and tergites dark brown; pedipalps reddish brown, paler in trochanter; chelicerae pale yellow. ***Carapace*** (Figs 5A, 6A): 1.24× longer than broad; surface smooth, but with a medial furrow; with two distinct eyespots situated near anterior margin of carapace; anterior margin with six setae, posterior margin with seven setae, 47 in total, each seta acicular and very slightly curved; with lyrifissures on both halves. ***Chelicera*** (Fig. 6B, D): much smaller than carapace length; surface smooth; four setae (sbs absent) and two lyrifissures (exterior condylar lyrifissure and exterior lyrifissure) present on hand; movable finger with one galeal seta (shorter than others); bs and es dentate apically, is and ls long and acute. Fixed finger with six large retrorse teeth and three small apical teeth, movable finger with two large teeth; galea present, slender and apically with five dentations (Fig. 6B). Serrula interior connected to fixed finger for entire length, proximally modified to form velum, serrula exterior with 27 or 29 lamellae, the basal one longest; lamina exterior present. Rallum composed of four blades, the basal two blades shorter than others, the distal one dentated anteriorly, remainder smooth (Fig. 6D). ***Pedipalp*** (Figs 5B–D, 6G, K): slightly stout, trochanter 1.80×, femur 2.46×, patella 2.02×, chela with pedicel (without pedicel) 2.68× (2.43×), hand with pedicel (without pedicel) 1.71× (1.46×) longer than broad; movable chelal finger 0.57× (0.67×) longer than hand with pedicel (without pedicel) and 0.37× (0.41×) longer than chela with pedicel (without pedicel). Setae generally long and acuminate. Retrolateral surface of trochanter and hand, prolateral surface of patella, femur, and hand granular; trochanter with a rounded ventral tubercle and a well-developed conical dorsal tubercle. Fixed chelal finger with eight trichobothria, movable chelal finger with four trichobothria: eb and esb situated at base of fixed finger on retrolateral face, esb slightly distal to eb; ib and isb situated at base of fixed finger on prolateral face, isb slightly distal to ib; est in the middle of fixed finger; et near sub-distal of fixed finger; est closer to et than to esb; it slightly distal to est and proximal to et; ist situated proximal to est and distal to isb; it closer to ist than to fingertip; distance between ist and isb twice that of ist and it; b and sb situated at base of movable finger on retrolateral face; t in the middle of movable finger and at same level as est; sb closer to b than to st; st midway between t and sb (Fig. 6G). Venom apparatus only present in fixed chelal finger, venom ducts curved and short, terminating in inflated nodus ramosus between et and est. Both chelal fingers with a row of acute teeth, spaced contiguously along the margin, slightly rounded proximally: fixed chelal finger with 26 or 28 teeth, with 12 sense spots between esb and est; movable chelal finger with 30 or 33 teeth (nearly as large as teeth on fixed chelal finger), basal half with ten sense spots; without accessory teeth (Fig. 6G). Femur without long tactile setae. Movable chelal finger slightly curved in lateral view (Figs 5B, 6G). ***Opisthosoma***: typical, all setae long and acuminate, setal bases distinct larger; pleural membrane longitudinally striate, without setae. Tergites I and XI undivided and other tergites incompletely divided, tergal chaetotaxy I–XI: 8: 8: 8: 12: 13: 7–8: 7–7: 8–7: 8–9: 8–8 (14 + 2T): 16 (14 + 2T). Sternites incompletely divided, sternal chaetotaxy IV–XI: 6–5: 8–9: 8–8: 7–7: 7–8: 8–8: 8–8 (14 + 2T): 14 (10 + 4T). Anus (tergite XII and sternite XII) without raised rim. Anterior genital operculum with 18 setae, posterior margin with nine setae. Male genitalia (Figs 5E, 6I, J): the distal part (l) of lateral apodemes with a distinctive inner ridge curved into semicircle; the hooked branch (br) bowed distally and terminated in a plate-like tip; the proximal part with a distinct dark sclerotized bar (c); the longitudinal fold of medial diverticula (d) with a projection midway along its length, forming a digitiform bulge; the ejaculatory canal atrium (e) not well-developed, curved distally; the lateral rods (f) long and diverging proximally; the tip of dorsal apodeme (g) completely joined; the ventral diverticulum (h) bilobed; genital atrium without genital setae. ***Legs*** (Figs 5G–I, 6E, F): typical, fairly smooth, slightly stout; junction between femora and patellae I and II oblique. Femoropatella of leg IV 2.74× longer than deep; tibia 3.43× longer than deep; with basal tactile setae on tarsal segment: tarsus 4.23× longer than deep (TS = 0.11); subterminal tarsal setae arcuate and acute. Arolium slightly shorter than claws, not divided; claws smooth.

**Adult females** (Figs 4B, 5F, 6C, H): mostly same as the male, but distinct larger; pedipalps color paler than that of male; carapace 1.17–1.23× longer than broad. ***Chelicera***: galea distinct large, with five large dentations and a bifurcate tip (Fig. 6C); serrula exterior with 21 lamellae. ***Pedipalp***: stout, trochanter 1.61–1.68×, femur 2.39–2.51×, patella 2.11–2.15×, chela (with pedicel) 2.54–2.57×, chela (without pedicel) 2.29–2.35×, hand (with pedicel) 1.65–1.69×, hand (without pedicel) 1.40–1.47× longer than broad, movable chelal finger 0.55–0.58× (0.63–0.68×) longer than hand with pedicel (without pedicel) and 0.36–0.38× (0.39–0.42×) longer than chela with pedicel (without pedicel). ***Opisthosoma***: genital structure (Figs 5F, 6H) simple, spermathecae provided with separated median cribriform plates; anterior genital operculum with 11 short setae, posterior margin with four marginal setae on each side, arranged in a row. ***Legs***: femoropatella of leg IV 2.73–2.75× longer than deep; tibia 3.30–3.71× longer than deep; with basal tactile setae on tarsal segment: tarsus 4.21–4.43× longer than deep (TS = 0.08).

***Dimensions*** (length/breadth or, in the case of the legs, length/depth in mm). Male (females in parentheses): body length 4.00 (5.72–5.86). Carapace 1.13/0.91 (1.23–1.29/1.05). Pedipalp: trochanter 0.63/0.35 (0.57–0.58/0.34–0.36), femur 1.01/0.41 (0.98/0.39–0.41), patella 1.01/0.50 (0.97–0.99/0.46), chela (with pedicel) 1.69/0.63 (1.73–1.75/0.68), chela (without pedicel) 1.53 (1.56–1.60), hand (with pedicel) 1.08 (1.12–1.15), hand (without pedicel) 0.92 (0.95–1.00), movable finger length 0.62 (0.63–0.65). Leg I: trochanter 0.21/0.20 (0.23–0.24/0.19–0.20), femur 0.33/0.26 (0.34–0.35/0.26), patella 0.54/0.24 (0.55–0.57/0.22–0.23), tibia 0.57/0.17 (0.58–0.59/0.17–0.18), tarsus 0.50/0.12 (0.51–0.52/0.11–0.12). Leg IV: trochanter 0.40/0.25 (0.41–0.42/0.27–0.28), femoropatella 0.96/0.35 (0.99–1.01/0.36–0.37), tibia 0.72/0.21 (0.76–0.78/0.21–0.23), tarsus 0.55/0.13 (0.59–0.62/0.14).

##### Remarks.

*Catatemnuslaminosus* sp. nov. can be distinguished from *C.birmanicus* by the trait of eyespots (with two distinct eyespots vs with two faint eyespots) and the traits of tergites (e.g., tergite III incompletely divided vs with trace of a division); from *C.concavus* by the trait of eyespots (with two distinct eyespots vs with two faint eyespots) and the traits of pedipalps (the dorsal surface of femur and hand almost smooth vs with minute and low granules everywhere except on fingers); from *C.monitor* by the trait of eyespots (with two distinct eyespots vs no eyespots visible) and the shorter legs (♂) (e.g., tibia of leg IV 3.43× vs 3.88× longer than broad, length 0.72 mm vs 0.84 mm; tarsus of leg IV 4.23× vs 5.00×, length 0.55 mm vs 0.63 mm); from *C.nicobarensis* by the trait of eyespots (with two distinct eyespots vs with two indistinct eyespots) and the traits of the palpal trochanter (anterior surface granular vs smooth or almost smooth); from *C.thorelli* by the trait of furrow on the carapace (distinctly curved vs almost straight) and the traits of pedipalps (surface of all segments (except chelal fingers) more or less granular vs surface of palps smooth; chelal hand (with pedicel) 1.71× vs 1.68× longer than broad, length 1.08 mm vs 1.25 mm) (Thorell 1889; Balzan 1891; With 1906; Beier 1932).

##### Distribution.

Known only from the type locality.

##### Etymology.

The specific name is derived from a Latin word *laminosus* (lamellar) and refers to the characters of serrula exterior with many lamellae.

#### ﻿ Catatemnus ramus sp. nov.

Taxon classificationAnimaliaPseudoscorpionesAtemnidae

F12745F9-1C38-56C6-BE3F-6F59970C158A

https://zoobank.org/A28D11D8-82F3-4571-8E33-46473B61B1BC

**Figure 7.**
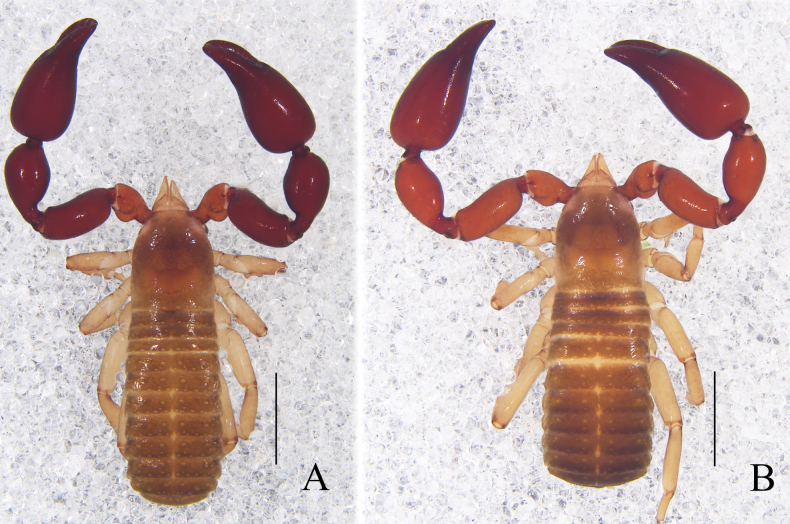
*Catatemnusramus* sp. nov. **A** holotype male, habitus (dorsal view) **B** paratype female, habitus (dorsal view). Scale bars: 1.00 mm.

**Figure 8.**
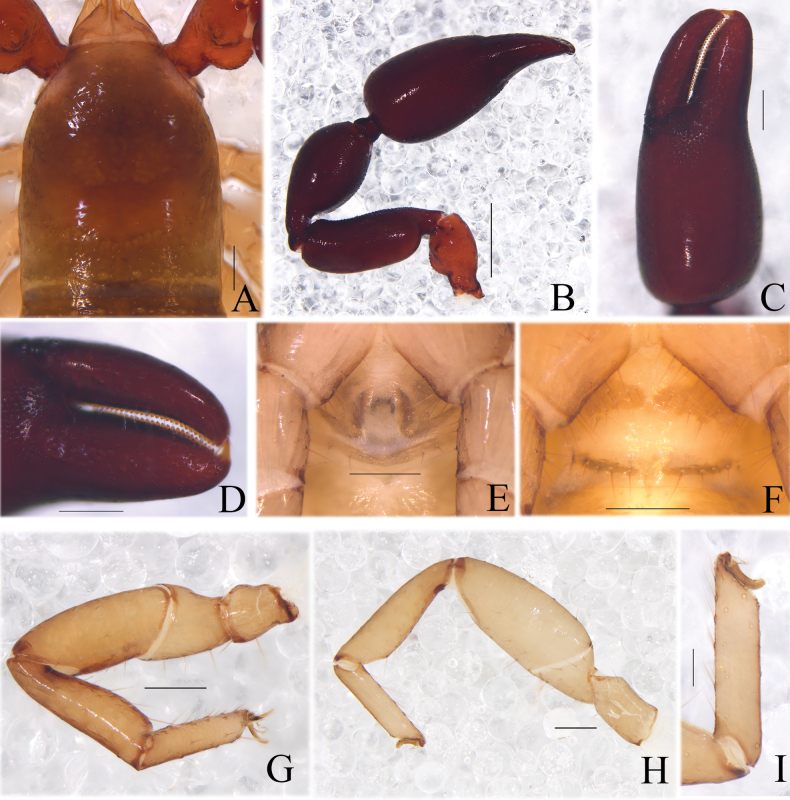
*Catatemnusramus* sp. nov., holotype male (**A–E, G–I**), paratype female (**F**) **A** carapace (dorsal view) **B** left pedipalp (dorsal view) **C** left chela (lateral view) **D** left chelal fingers (lateral view) **E** male genital area (ventral view) **F** female genital area (ventral view) **G** left leg I (lateral view) **H** left leg IV (lateral view) **I** tarsus of left leg IV (lateral view). Scale bars: 0.50 mm (**B**); 0.20 mm (**A, C–H**); 0.10 mm (**I**).

**Figure 9.**
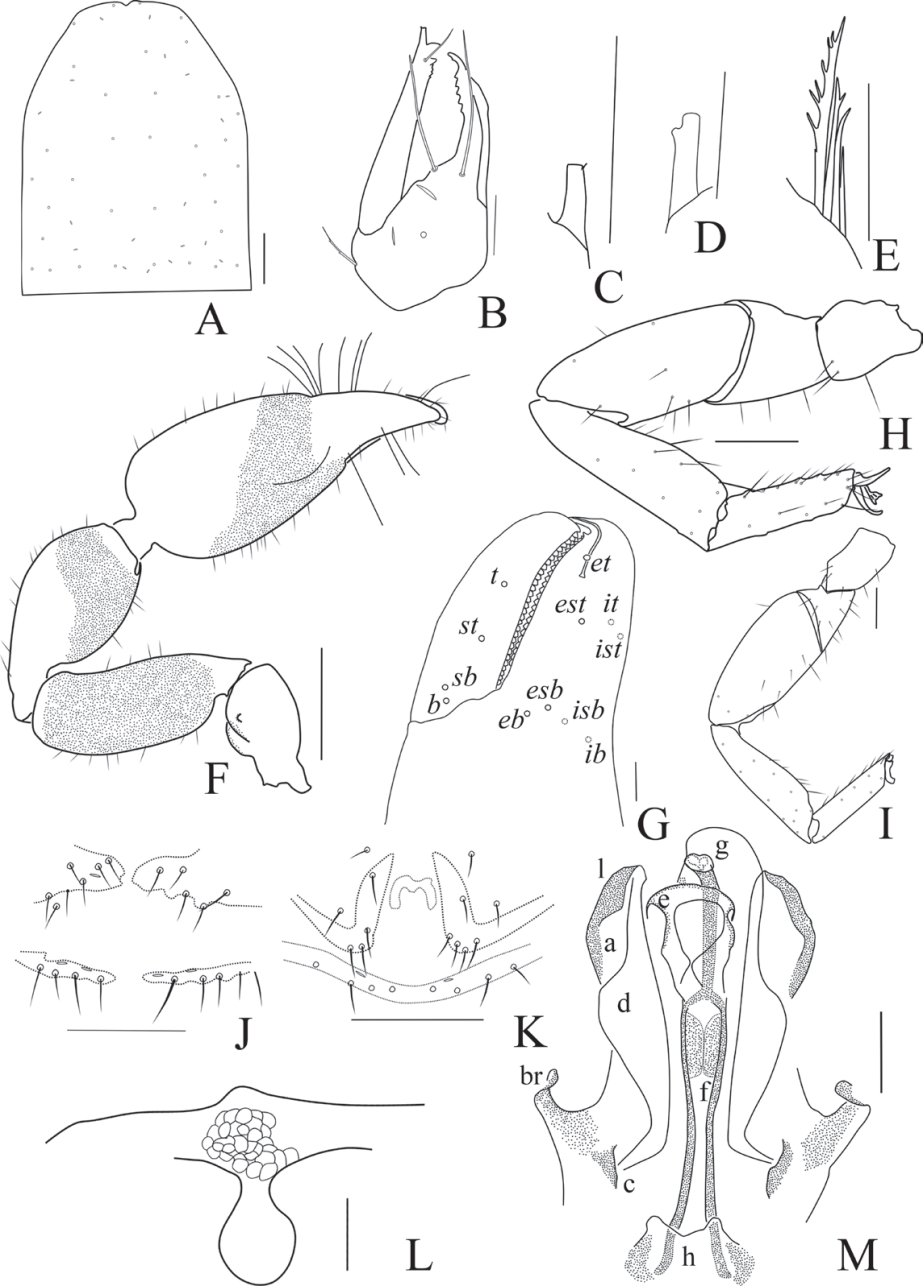
*Catatemnusramus* sp. nov., holotype male (**A–C, E–I, K, M**), paratype female (**D, J, L**) **A** carapace (dorsal view) **B** left chelicera (dorsal view) **C** male galea **D** female galea **E** rallum **F** left pedipalp (dorsal view) **G** left chelal fingers (lateral view), with details of trichobothrial pattern **H** left leg I (lateral view) **I** left leg IV (lateral view) **J** female genital area (ventral view) **K** male genital area (ventral view) **L** spermatheca **M** male genital organ. Scale bars: 0.50 mm (**F**); 0.20 mm (**A, H–K**); 0.10 mm (**B–E, G, L, M**). Abbreviations: **b** = basal; **sb** = sub-basal; **st** = sub-terminal; **t** = terminal; **ib** = interior basal; **isb** = interior sub-basal; **ist** = interior sub-terminal; **it** = interior terminal; **eb** = exterior basal; **esb** = exterior sub-basal; **est** = exterior sub-terminal; **et** = exterior terminal; **a** = lateral apodeme; **br** = hooked branch; **c** = sclerotized bar; **d** = longitudinal fold of medial diverticulum; **e** = ejaculatory canal atrium; **f** = lateral rods; **g** = dorsal apodeme; **h** = ventral diverticulum; **l** = lateral lip of lateral apodeme.

Figs 7 , 8 , 9 (枝猫伪蝎)

##### Type material.

***Holotype***: China • ♂; Yunnan Province, Longchuan County, Huguo Township; 24°33.682'N, 98°3.735'E; 1820 m a.s.l.; 09 Aug. 2013; Guanglin Xie leg.; Ps.–MHBU–YNLC13080901. ***Paratypes***: • 2♂5♀; same data as the holotype; Ps.–MSWU–YNLC13080902–13080908.

##### Diagnosis (♂♀).

This new species is characterized by the rallum with two dentated blades instead of the usual one; with small body size (♂) 2.94–3.23 mm, (♀) 3.13–3.69 mm; carapace smooth but with a medial furrow; with two distinct, developed, eyespots situated near anterior margin of carapace. Palpal femur (♂) 2.59–2.76×, (♀) 2.50–2.51×, chela with pedicel (♂) 2.49–2.66×, (♀) 2.37–2.49×, chela without pedicel (♂) 2.31–2.45×, (♀) 2.25–2.35× longer than broad.

##### Description.

**Adult males** (Figs 7A, 8A–E, G–I, 9A–C, E–I, K, M). ***Color***: anterior half of carapace yellowish brown, paler in posterior half; pedipalps reddish brown, paler in female; tergites brown, paler in legs; remainder yellow. ***Carapace*** (Figs 8A, 9A): 1.25× longer than broad; surface smooth, but with a medial furrow; with two distinct eyespots situated near anterior margin of carapace; anterior margin with four setae, posterior margin with eight or nine setae, 34 or 35 in total, each seta acicular and very slightly curved; more lyrifissures on posterior margin. ***Chelicera*** (Figs 9B, C, E): much smaller than carapace length; surface smooth; four setae (sbs absent) and two lyrifissures (exterior condylar lyrifissure and exterior lyrifissure) present on hand; movable finger with one galeal seta (shorter than others); bs and es dentate apically, is and ls long and acute. Fixed finger with five large retrorse teeth and two or three small apical teeth, movable finger with one large teeth; galea present, shorter and with seven or eight branchlets (Fig. 9C). Serrula interior connected to fixed finger for entire length, proximally modified to form velum, serrula exterior with 26 lamellae, the basal one longest; lamina exterior present. Rallum composed of four blades, the basal two blades shorter than others, the distal two dentated anteriorly, remainder smooth (Fig. 9E). ***Pedipalp*** (Figs 8B–D, 9F, G): stout, trochanter 1.52–1.59×, femur 2.59–2.76×, patella 1.93–1.94×, chela with pedicel (without pedicel) 2.49–2.66× (2.31–2.45×), hand without pedicel 1.37–1.44× longer than broad; movable chelal finger 0.56–0.61× (0.63–0.71×) longer than hand with pedicel (without pedicel). Setae generally long and acuminate. Retrolateral surface of trochanter, prolateral surface of patella, surface of femur and hand granular; trochanter with a rounded ventral tubercle and a well-developed conical dorsal tubercle. Fixed chelal finger with eight trichobothria, movable chelal finger with four trichobothria: eb and esb situated at base of fixed finger on retrolateral face, esb slightly distal to eb; ib and isb situated at base of fixed finger on prolateral face, isb slightly distal to ib; est in the middle of fixed finger; et near sub-distal of fixed finger; est closer to et than to esb; it slightly distal to est and proximal to et; ist situated proximal to est and distal to isb; it closer to ist than to fingertip; distance between est and esb nearly equal to that of ist and isb; b and sb situated at base of movable finger on retrolateral face; t in the middle of movable finger and at same level as est; sb closer to b than to st; st midway between t and sb (Fig. 9G). Venom apparatus only present in fixed chelal finger, venom ducts curved and short, terminating in inflated nodus ramosus between et and est, closer to et. Both chelal fingers with a row of acute teeth, spaced contiguously along the margin, slightly rounded proximally: fixed chelal finger with 25 teeth; movable chelal finger with 32 teeth (nearly as large as teeth on fixed chelal finger), basal half with ten sense spots; without accessory teeth (Fig. 9G). Femur without long tactile setae. Movable chelal finger slightly curved in lateral view (Figs 8D, 9G). ***Opisthosoma***: typical, all setae long and acuminate, setal bases distinct larger; pleural membrane longitudinally striate, without setae. Tergites I and XI undivided and other tergites incompletely divided, tergal chaetotaxy I–XI: 8: 8: 9: 8: 15: 16: 17: 16: 17: 15+ (2T): 10+ (4T). Sternites XI undivided and other sternites divided, sternal chaetotaxy IV–XI: 7–8: 9–7: 8–7: 7–7: 8–9: 7–6 (4T): 11 + (4T). Anus (tergite XII and sternite XII) without raised rim. Anterior genital operculum with 12 setae, posterior margin with eight setae on each side and one or two lyrifissures. Male genitalia (Figs 8E, 9K, M): the distal part (l) of lateral apodemes with a distinctive inner ridge curved into semicircle; the hooked branch (br) bowed distally and terminated in a plate-like tip; the proximal part with a distinct dark sclerotized bar (c); the longitudinal fold of medial diverticula (d) with a projection midway along its length, forming a lobe bulge; the ejaculatory canal atrium (e) not well-developed, curved distally; the lateral rods (f) long and diverging proximally; the tip of dorsal apodeme (g) completely joined; the ventral diverticulum (h) bilobed; genital atrium without genital setae. ***Legs*** (Figs 8G–I, 9H, I): typical, fairly smooth, slightly stout; junction between femora and patellae I and II oblique. Femoropatella of leg IV 2.82–2.83× longer than deep; tibia 3.55–3.80× longer than deep; with basal tactile setae on tarsal segment: tarsus 4.14–4.15× longer than deep (TS = 0.09–0.11); subterminal tarsal setae arcuate and acute. Arolium slightly longer than claws, not divided; claws smooth.

**Adult females** (Figs 7B, 8F, 9D, J, L): mostly same as the male, but a litter larger in body size; anterior half of carapace yellowish brown, paler in posterior half; pedipalps reddish brown, paler, and paler from fingertip to trochanter; tergites yellowish brown, paler in legs. ***Chelicera***: hand with four setae; fixed finger with four large retrorse teeth and two or three small apical teeth, movable finger with a long, broadly rounded, subapical lobe; galea distinct relatively short, with seven or eight branchlets (Fig. 9D); serrula exterior with 24 lamellae; rallum composed of four blades, the basal two blades shorter than others, the distal two dentated anteriorly, remainder smooth. ***Pedipalp***: stout, trochanter 1.56–1.74×, femur 2.50–2.51×, patella 1.96–2.06×, chela (with pedicel) 2.37–2.49×, chela (without pedicel) 2.25–2.35×, hand (without pedicel) 1.49–1.56× longer than broad, movable chelal finger 0.56–0.55× (0.59–0.61×) longer than hand with pedicel (without pedicel).

**Dimensions** (length/breadth or, in the case of the legs, length/depth in mm). Males (females in parentheses): body length 2.94–3.23 (3.13–3.69). Carapace 1.09–1.14/0.87–0.91 (1.16–1.20/0.91–0.95). Pedipalp: trochanter 0.50–0.54/0.33–0.34 (0.56–0.61/0.35–0.36), femur 0.96–1.02/0.37 (1.00–1.03/0.40–0.41), patella 0.87–0.93/0.45–0.48 (0.90–0.99/0.46–0.48), chela (with pedicel) 1.52–1.65/0.61–0.62 (1.68–1.72/0.69–0.71), chela (without pedicel) 1.41–1.52 (1.60–1.62), hand (with pedicel) 0.98–0.99 (1.13–1.19), hand (without pedicel) 0.85–0.88 (1.03–1.11), movable finger length 0.55–0.60 (0.63–0.66). Leg I: trochanter 0.21–0.22/0.19–0.20 (0.41–0.43/0.27–0.29), femur 0.31–0.35/0.24 (0.33–0.36/0.19–0.22), patella 0.50–0.56/0.21–0.24 (0.53–0.56/0.21–0.23), tibia 0.53–0.59/0.16–0.17 (0.51–0.61/0.15–0.17), tarsus 0.51–0.53/0.11 (0.46–0.53/0.10–0.12). Leg IV: trochanter 0.39–0.48/0.22–0.26 (0.44–0.45/0.24–0.29), femoropatella 0.96–1.02/0.34–0.36 (1.00–1.02/0.36–0.37), tibia 0.76–0.78/0.20–0.22 (0.80–0.83/0.21–0.22), tarsus 0.54–0.58/0.13–0.14 (0.56–0.57/0.14).

##### Remarks.

*Catatemnusramus* sp. nov. can be distinguished from *C.birmanicus* by the smaller body size (e.g., body length (♂) 2.94–3.23 mm vs 4.27 mm, (♀) 3.13–3.69 mm vs 4.62 mm; palpal femur (♂) 2.59–2.76× vs 2.44×, (♀) 2.50–2.51× vs 2.36× longer than broad, length (♂) 0.96–1.02 mm vs 1.09 mm, (♀) 1.00–1.03 mm vs 0.92 mm) and the trait of eyespots (with two distinct eyespots vs with two faint eyespots); from *C.concavus* by the trait of eyespots (with two distinct eyespots vs with two faint eyespots) and the slender pedipalps (♀) (e.g., palpal femur 2.50–2.51× vs 2.29× longer than broad); from *C.monitor* by the trait of eyespots (with two distinct eyespots vs no eyespots visible), the trait of rallum (rallum with two dentated blades vs with one dentated blade only) and the traits of pedipalps (distinctly granular vs minutely granular); from *C.nicobarensis* by the trait of eyespots (with two distinct eyespots vs with two indistinct eyespots) and the traits of the palpal trochanter (anterior surface granular vs smooth or almost smooth); from *C.thorelli* by the trait of furrow on the carapace (distinctly curved vs almost straight) and the traits of pedipalps (surface of all segments (except chelal fingers) granular vs surface of palps smooth; palpal femur 2.59–2.76× vs 2.40× longer than broad, length 0.96–1.02 mm vs 1.15 mm; movable finger teeth length 0.55–0.60 mm vs 0.70 mm) (Thorell 1889; Balzan 1891; With 1906; Beier 1932).

##### Distribution.

Known only from the type locality.

##### Etymology.

The specific name is derived from a Latin word *ramus* (branched) and refers to the characters of the rallum with two dentate blades.

#### ﻿ Catatemnus scaber sp. nov.

Taxon classificationAnimaliaPseudoscorpionesAtemnidae

5BA0B899-E756-5AA9-852F-35CCF22EC2E1

https://zoobank.org/84384592-433C-4CF6-9093-5C519173C6FA

**Figure 10.**
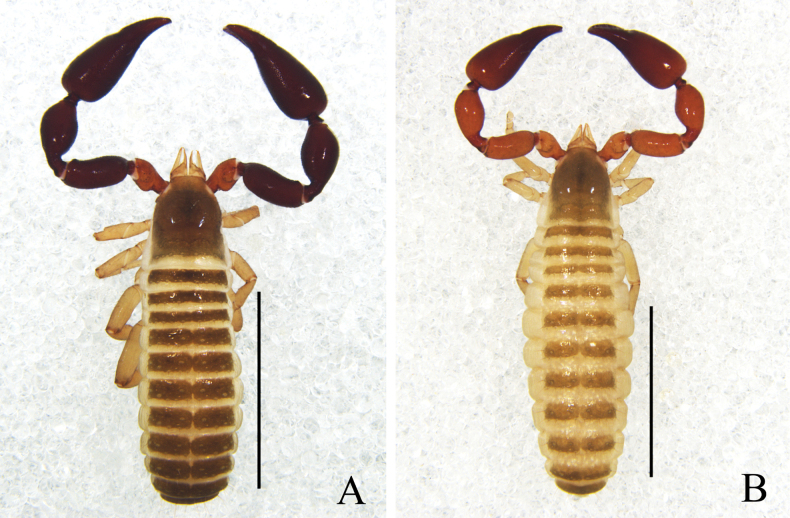
*Catatemnusscaber* sp. nov. **A** holotype male, habitus (dorsal view) **B** paratype female, habitus (dorsal view). Scale bars: 2.00 mm.

**Figure 11.**
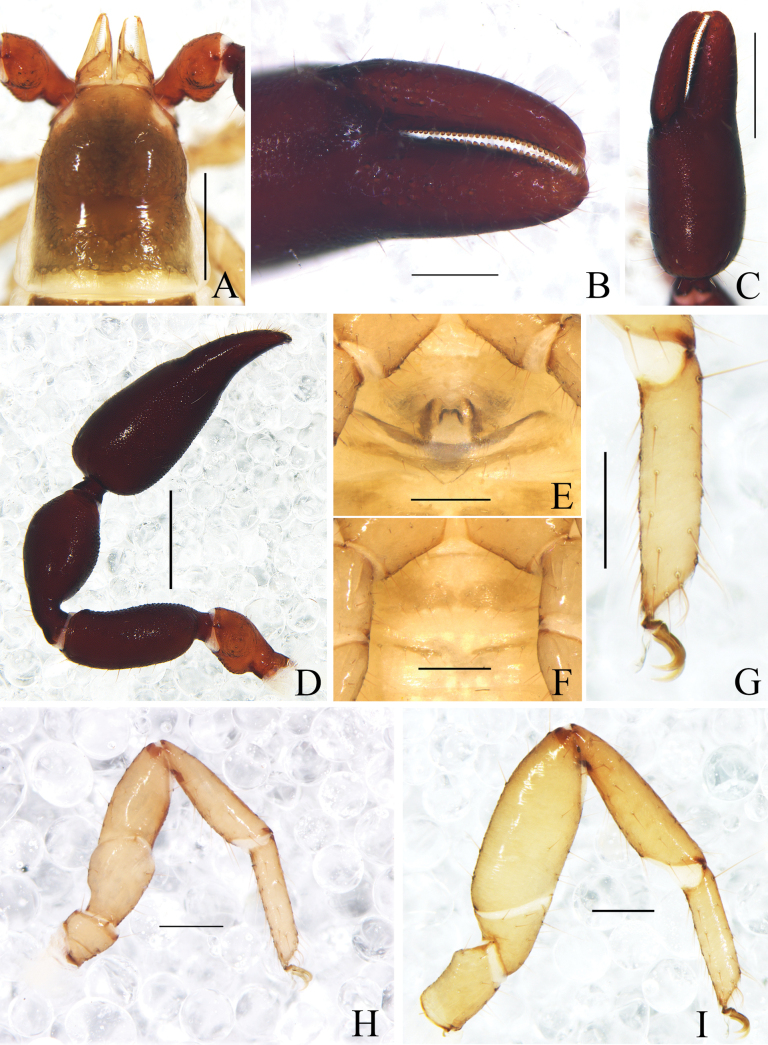
*Catatemnusscaber* sp. nov., holotype male (**A–E, G–I**), paratype female (**F**) **A** carapace (dorsal view) **B** left chelal fingers (lateral view) **C** left chela (lateral view) **D** left pedipalp (dorsal view) **E** male genital area (ventral view) **F** female genital area (ventral view) **G** tarsus of left leg IV (lateral view) **H** left leg I (lateral view) **I** left leg IV (lateral view). Scale bars: 0.50 mm (**A, C, D**); 0.20 mm (**B, E–I**).

**Figure 12.**
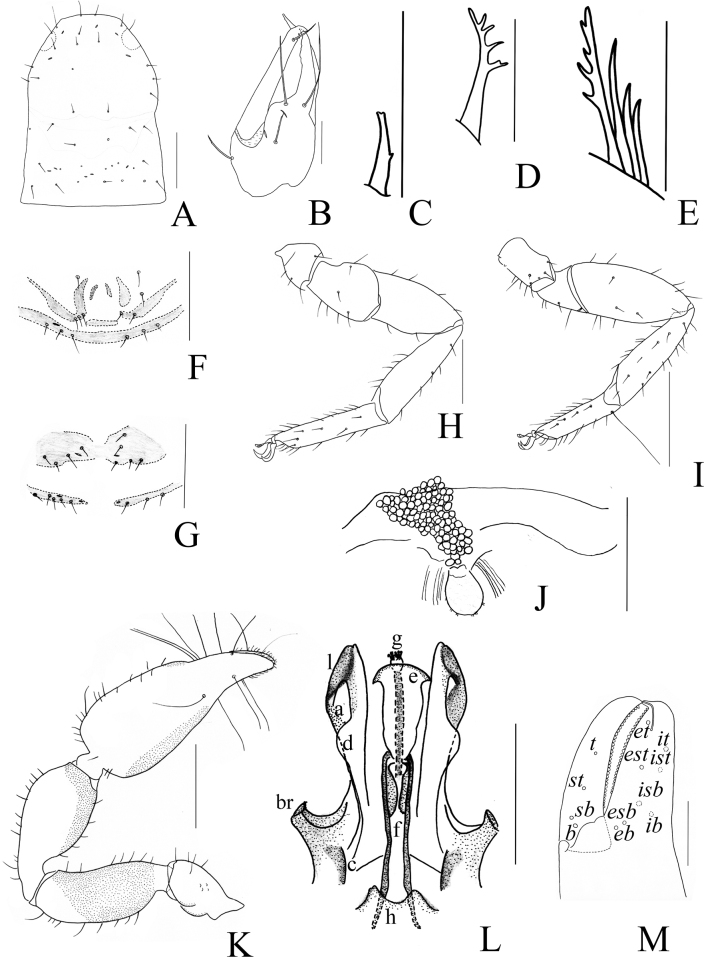
*Catatemnusscaber* sp. nov., holotype male (**A–C, E, F, H, I, K–M**), paratype female (**D, G, J**) **A** carapace (dorsal view) **B** left chelicera (dorsal view) **C** male galea **D** female galea **E** rallum **F** male genital area (ventral view) **G** female genital area (ventral view) **H** left leg I (lateral view) **I** left leg IV (lateral view) **J** spermatheca **K** left pedipalp (dorsal view) **L** male genital organ **M** left chelal fingers (lateral view), with details of trichobothrial pattern. Scale bars: 0.50 mm (**I, K**); 0.25 mm (**A, F–H, J, L, M**); 0.10 mm (**B–E**). Abbreviations: **b** = basal; **sb** = sub-basal; **st** = sub-terminal; **t** = terminal; **ib** = interior basal; **isb** = interior sub-basal; **ist** = interior sub-terminal; **it** = interior terminal; **eb** = exterior basal; **esb** = exterior sub-basal; **est** = exterior sub-terminal; **et** = exterior terminal; **a** = lateral apodeme; **br** = hooked branch; **c** = sclerotized bar; **d** = longitudinal fold of medial diverticulum; **e** = ejaculatory canal atrium; **f** = lateral rods; **g** = dorsal apodeme; **h** = ventral diverticulum; **l** = lateral lip of lateral apodeme.

Figs 10 , 11 , 12 (粗糙猫伪蝎)

##### Type material.

***Holotype***: China • ♂; Yunnan Province, Longyang District, Lujiang Town, Bawan Village; 24°56.871'N, 98°49.969'E; 1025 m a.s.l.; 29 May. 2017; Chi Jin, Beibei Zhou and Xiao Zang leg.; Ps.–MHBU–YNBS170101. ***Paratypes***: • 8♂10♀; same data as the holotype; Ps.–MSWU–YNBS170102–19.

##### Diagnosis (♂♀).

This new species is characteristic by: palpal femur (♂) 2.38–2.65×, (♀) 2.44–2.50×, chela with pedicel (♂) 2.76–2.83×, (♀) 2.61×, without pedicel (♂) 2.51–2.60×, (♀) 1.57–1.65× longer than broad; distance between est and esb farther to that of ist and isb, st midway between sb and t; male genitalia: the longitudinal fold of medial diverticula (d) with a projection midway along its length, forming a lobe bulge.

##### Description.

**Adult males** (Figs 10A, 11A–E, G–I, 12A–C, E, F, H, I, K–M). ***Color***: carapace and tergites dark brown; pedipalps reddish brown, but paler in trochanter; legs pale yellow. ***Carapace*** (Figs 11A, 12A): 1.16–1.33× longer than broad; surface smooth, but with a medial furrow; with two distinct eyespots situated near anterior margin of carapace; anterior margin with six setae, posterior margin with eight setae, each seta acicular and very slightly curved. ***Chelicera*** (Fig. 12B, C, E): much smaller than carapace length; surface smooth; four setae (sbs absent) and two lyrifissures (exterior condylar lyrifissure and exterior lyrifissure) present on hand; movable finger with one galeal seta (shorter than others); bs and es dentate apically, is and ls long and acute. Fixed finger with five large retrorse teeth and two or three small apical teeth, movable finger with one large teeth; galea present, shorter and with seven or eight branchlets (Fig. 12C). Serrula interior connected to fixed finger for entire length, proximally modified to form velum, serrula exterior with 26 lamellae, the basal one longest; lamina exterior present. Rallum composed of four blades, the basal two blades shorter than others, the distal two dentated anteriorly, remainder smooth (Fig. 12E). ***Pedipalp*** (Figs 11B–D, 12K, M): stout, trochanter 1.62–1.79×, femur 2.38–2.65×, patella 2.08–2.11×, chela with pedicel (without pedicel) 2.76–2.83× (2.51–2.60×), hand with pedicel (without pedicel) 1.65–1.73× (1.41–1.50×) longer than broad; movable chelal finger 0.64–0.67× (0.74–0.78×) longer than hand with pedicel (without pedicel) and 0.39–0.40× (0.42–0.44×) longer than chela with pedicel (without pedicel). Setae generally long and acuminate. Retrolateral surface of trochanter, surface of femur and hand granular; trochanter with two inconspicuous tubercles. Fixed chelal finger with eight trichobothria, movable chelal finger with four trichobothria: eb and esb situated at base of fixed finger on retrolateral face, esb slightly distal to eb; ib and isb situated at base of fixed finger on prolateral face, isb slightly distal to ib; est in the middle of fixed finger; et near sub-distal of fixed finger; est closer to et than to esb; it slightly distal to est and proximal to et; ist situated proximal to est and distal to isb; it closer to ist than to fingertip; distance between est and esb farther to that of ist and isb; distance between it and fingertip shorter to distance between ist and isb; b and sb situated at base of movable finger on retrolateral face; t in the middle of movable finger and at same level as est; sb closer to b than to st; st midway between t and sb (Fig. 12M). Venom apparatus only present in fixed chelal finger, venom ducts curved and short, terminating in inflated nodus ramosus between et and est, closer to et. Both chelal fingers with a row of acute teeth, spaced contiguously along the margin, slightly rounded proximally: fixed chelal finger with 23 or 25 teeth; movable chelal finger with 32 teeth (nearly as large as teeth on fixed chelal finger); without accessory teeth (Fig. 12M). Femur without long tactile setae. Movable chelal finger slightly curved in lateral view (Figs 11B, 12M). ***Opisthosoma***: typical, all setae long and acuminate, setal bases distinct larger; pleural membrane longitudinally striate, without setae. Tergites I–III and XI undivided and other tergites incompletely divided, tergites I–III each with nine setae, uniseriate; other tergites with 13 setae, biseriate; tergites X and XI each with a pair of tactile setae. Sternites completely divided, each half sternites with seven or eight setae; sternites X and XI each with two pairs of long tactile setae. Anus (tergite XII and sternite XII) without raised rim. Anterior genital operculum with 18 setae. Male genitalia (Figs 11E, 12F, L): the distal part (l) of lateral apodemes with a distinctive inner ridge curved into semicircle; the hooked branch (br) bowed distally and terminated in a plate-like tip; the proximal part with a distinct dark sclerotized bar (c); the longitudinal fold of medial diverticula (d) with a projection midway along its length, forming a lobe bulge; the ejaculatory canal atrium (e) not well-developed, curved distally; the lateral rods (f) long and diverging proximally; the tip of dorsal apodeme (g) completely joined; the ventral diverticulum (h) bilobed; genital atrium without genital setae. ***Legs*** (Figs 11G–I, 12H, I): typical, fairly smooth, slightly stout; junction between femora and patellae I and II oblique. Femoropatella of leg IV 2.86–3.07× longer than deep; tibia 3.47–3.71× longer than deep; with basal tactile setae on tarsal segment: tarsus 4.09–4.18× longer than deep (TS = 0.11); subterminal tarsal setae arcuate and acute. Arolium slightly shorter than claws, not divided; claws smooth.

**Adult females** (Figs 10B, 11F, 12G, J): mostly same as the male, but a little larger in body size and paler; pedipalps paler, reddish brown, and paler from fingertip to trochanter; carapace 1.16–1.25× longer than broad. ***Chelicera***: hand with four setae, gs shorter than others; galea distinct relatively short, with ten branchlets (Fig. 12D); serrula exterior with 20 or 21 lamellae; rallum composed of four blades, the distal one dentate anteriorly. ***Pedipalp***: stout, trochanter 1.57–1.62, femur 2.44–2.50, patella 2.11–2.14, chela (with pedicel) 2.61, chela (without pedicel) 2.41–2.43, hand (with pedicel) 1.57–1.65, hand (without pedicel) 1.39–1.45× longer than broad, movable chelal finger 0.60–0.65× (0.69–0.73×) longer than hand with pedicel (without pedicel) and 0.38–0.39× (0.41–0.42×) longer than chela with pedicel (without pedicel). ***Opisthosoma***: genitalia simple, spermathecae provided with separated median cribriform plates; anterior genital operculum with ten setae. ***Legs***: femoropatella of leg IV 2.79–2.89× longer than deep; tibia 3.33–3.47× longer than deep; with basal tactile setae on tarsal segment: tarsus 3.75–4.00× longer than deep (TS = 0.11).

**Dimensions** (length/breadth or, in the case of the legs, length/depth in mm). Males (females in parentheses): body length 2.99–3.35 (4.11–4.64). Carapace 0.93–0.96/0.72–0.80 (0.95–1.00/0.80–0.82). Pedipalp: trochanter 0.47–0.52/0.29 (0.44–0.47/0.28–0.29), femur 0.76–0.82/0.31–0.32 (0.75–0.78/0.30–0.32), patella 0.80–0.83/0.38–0.40 (0.77–0.78/0.36–0.37), chela (with pedicel) 1.35–1.36/0.48–0.49 (1.33–1.41/0.51–0.54), chela (without pedicel) 1.23–1.25 (1.23–1.31), hand (with pedicel) 0.81–0.83 (0.84–0.85), hand (without pedicel) 0.69–0.72 (0.74–0.75), movable finger length 0.53–0.54 (0.50–0.55). Leg I: trochanter 0.17–0.19/0.14–0.16 (0.16–0.18/0.14–0.15), femur 0.28–0.29/0.20–0.21 (0.25–0.26/0.20), patella 0.42–0.43/0.18–0.19 (0.41/0.18), tibia 0.42–0.45/0.13 (0.42–0.43/0.12–0.13), tarsus 0.40–0.42/0.09 (0.35–0.38/0.10–0.11). Leg IV: trochanter 0.32–0.33/0.19–0.22 (0.34/0.21), femoropatella 0.80–0.83/0.27–0.28 (0.81/0.28–0.29), tibia 0.59–0.63/0.17 (0.59–0.60/0.17–0.18), tarsus 0.45–0.46/0.11 (0.44–0.45/0.11–0.12).

##### Remarks.

*Catatemnusscaber* sp. nov. can be distinguished from *C.birmanicus* by the smaller body size (♂) (e.g., body length 2.99–3.35 mm vs 4.27 mm; palpal femur length 0.76–0.82 mm vs 1.09 mm; movable chelal finger length 0.53–0.54 mm vs 0.78 mm) and the trait of eyespots (with two distinct eyespots vs with two faint eyespots); from *C.concavus* by the trait of eyespots (with two distinct eyespots vs with two faint eyespots), the stouter pedipalps (♀) (palpal femur 2.44–2.50× vs 2.29× longer than broad, length 0.75–0.78 mm vs 0.90 mm) and the traits of the three first tergites (regularly convex from the one side to the other vs with a descending lateral portion, perpendicular on the dorsal part); from *C.monitor* by the trait of eyespots (with two distinct eyespots vs no eyespots visible) and the stouter pedipalps (e.g., palpal femur (♂) 2.38–2.65× vs 2.25×, (♀) 2.44–2.50× vs 2.27× longer than broad, length (♂) 0.76–0.82 mm vs 1.01 mm, (♀) 0.75–0.78 mm vs 0.95 mm); from *C.nicobarensis* by the trait of eyespots (with two distinct eyespots vs with two indistinct eyespots) and the traits of the palpal trochanter (anterior surface granular vs smooth or almost smooth); from *C.thorelli* by the traits of pedipalps (surface of all segments except chelal fingers granular vs surface of palps smooth; palpal femur 2.38–2.65× vs 2.40× longer than broad, length 0.76–0.82 mm vs 1.15 mm; movable finger teeth length 0.53–0.54 mm vs 0.70 mm) (Thorell 1889; Balzan 1891; With 1906; Beier 1932).

##### Distribution.

Known only from the type locality.

##### Etymology.

The specific name is derived from the Latin word *scaber* (rough) and refers to the large number of granulations present on the surface of the pedipalp.

#### ﻿ Catatemnus tengchongensis sp. nov.

Taxon classificationAnimaliaPseudoscorpionesAtemnidae

11FBD265-6E4F-5192-BB03-F0DCB46C4619

https://zoobank.org/344BB985-9DD6-4CBC-96A8-86B315B3F1AE

**Figure 13.**
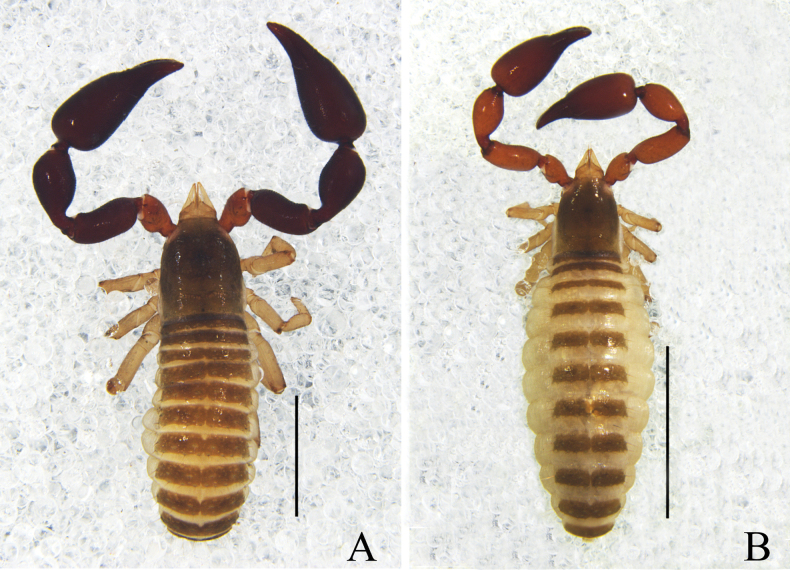
*Catatemnustengchongensis* sp. nov. **A** holotype male, habitus (dorsal view) **B** paratype female, habitus (dorsal view). Scale bars: 2.00 mm (**B**); 1.00 mm (**A**).

**Figure 14.**
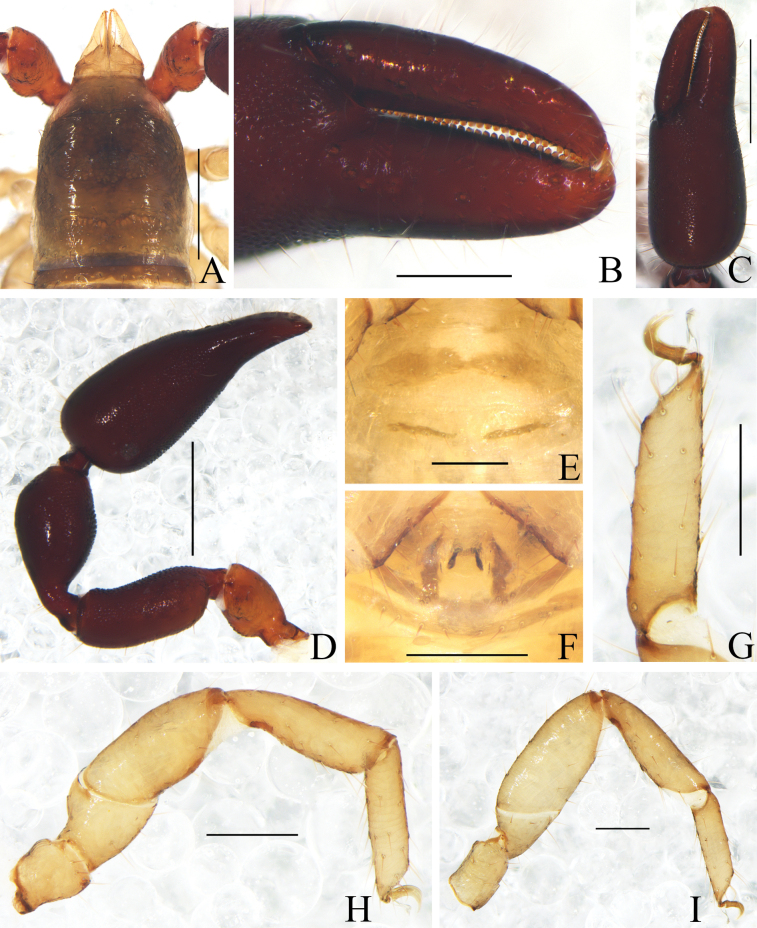
*Catatemnustengchongensis* sp. nov., holotype male (**A–D, F–I**), paratype female (**E**) **A** carapace (dorsal view) **B** left chelal fingers (lateral view) **C** left chela (lateral view) **D** left pedipalp (dorsal view) **E** female genital area (ventral view) **F** male genital area (ventral view) **G** tarsus of left leg IV (lateral view) **H** left leg I (lateral view) **I** left leg IV (lateral view). Scale bars: 0.50 mm (**A, C, D**); 0.20 mm (**B, E–I**).

**Figure 15.**
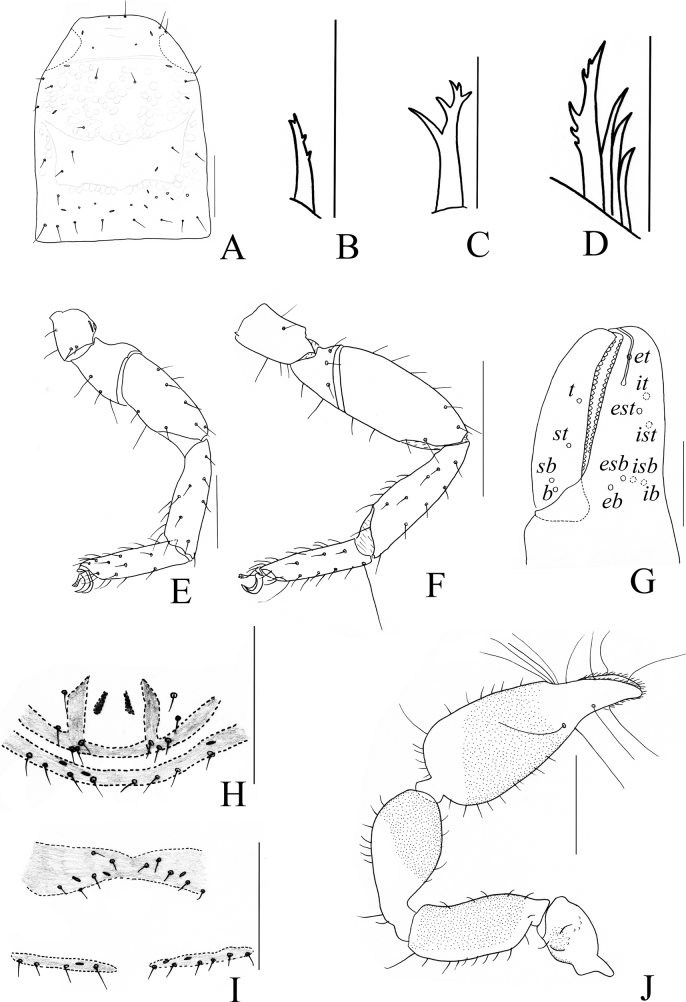
*Catatemnustengchongensis* sp. nov., holotype male (**A, B, D–H, J**), paratype female (**C, I**) **A** carapace (dorsal view) **B** male galea **C** female galea **D** rallum **E** left leg I (lateral view) **F** left leg IV (lateral view) **G** left chelal fingers (lateral view), with details of trichobothrial pattern **H** male genital area (ventral view) **I** female genital area (ventral view) **J** left pedipalp (dorsal view). Scale bars: 0.50 mm (**F, J**); 0.25 mm (**A, E, G–I**); 0.10 mm (**B–D**). Abbreviations: **b** = basal; **sb** = sub-basal; **st** = sub-terminal; **t** = terminal; **ib** = interior basal; **isb** = interior sub-basal; **ist** = interior sub-terminal; **it** = interior terminal; **eb** = exterior basal; **esb** = exterior sub-basal; **est** = exterior sub-terminal; **et** = exterior terminal.

Figs 13 , 14 , 15 (腾冲猫伪蝎)

##### Type material.

***Holotype***: China • ♂; Yunnan Province, Tengchong City, Houqiao Town, Yangjiatian Village; 25°21.413'N, 98°15.308'E; 1824 m a.s.l.; 07 Jun. 2017; Chi Jin, Beibei Zhou and Xiao Zang leg.; Ps.–MHBU–YNTC171201. ***Paratypes***: • 3♀; same data as the holotype; Ps.–MSWU–YNTC171202–04.

##### Diagnosis (♂♀).

This new species is characterized by: the serrula exterior with 25 lamellae; movable chelal finger with 28 teeth; palpal femur (♂) 2.59×, (♀) 2.42–2.52×, chela with pedicel (♂) 2.72×, (♀) 2.57–2.67×, chela without pedicel (♂) 2.51×, (♀) 2.37–2.42× longer than broad; distance between est and esb farther to that of ist and isb.

##### Description.

**Adult male** (Figs 13A, 14A–D, F–I, 15A, B, D–H, J). ***Color***: carapace dark brown, slightly paler in tergites and sternites; pedipalps reddish brown, but paler in trochanter; legs pale yellow. ***Carapace*** (Figs 14A, 15A): 1.20× longer than broad; surface smooth, but with a medial furrow; with two distinct eyespots situated near anterior margin of carapace; anterior margin with five setae, posterior margin with eight setae, each seta acicular and very slightly curved. ***Chelicera*** (Fig. 15B, D): much smaller than carapace length; surface smooth; four setae (sbs absent; es shorter than others) and two lyrifissures (exterior condylar lyrifissure and exterior lyrifissure) present on hand; movable finger with one galeal seta (shorter than others); bs and es dentate apically, is and ls long and acute. Fixed finger with seven unequally sized teeth, movable finger with two large teeth; galea present, shorter with two small branchlets and two dentations (Fig. 15B). Serrula interior connected to fixed finger for entire length, proximally modified to form velum, serrula exterior with 25 lamellae, the basal one longest; lamina exterior present. Rallum composed of four blades, the basal two blades shorter than others, the distal two dentated anteriorly, remainder smooth (Fig. 15D). ***Pedipalp*** (Figs 14B–D, 15G, J): stout, trochanter 1.77×, femur 2.59×, patella 1.95×, chela with pedicel (without pedicel) 2.72× (2.51×), hand with pedicel (without pedicel) 1.66× (1.45×) longer than broad; movable chelal finger 0.64× (0.74×) longer than hand with pedicel (without pedicel) and 0.39× (0.42×) longer than chela with pedicel (without pedicel). Setae generally long and acuminate. All segments (except chelal fingers) with fine granulations; trochanter with an inconspicuous ventral tubercle and a well-developed conical dorsal tubercle. Fixed chelal finger with eight trichobothria, movable chelal finger with four trichobothria: eb and esb situated at base of fixed finger on retrolateral face, esb slightly distal to eb; ib and isb situated at base of fixed finger on prolateral face, isb slightly distal to ib; est in the middle of fixed finger; et near sub-distal of fixed finger; est midway between et and esb; it slightly distal to est and proximal to et; ist situated proximal to est and distal to isb; it closer to ist than to fingertip; distance between est and esb farther to that of ist and isb; distance between it and fingertip nearly equal to distance between ist and isb; b and sb situated at base of movable finger on retrolateral face; t in the middle of movable finger and at same level as est; sb closer to b than to st; st closer to sb than to t (Fig. 15G). Venom apparatus only present in fixed chelal finger, venom ducts curved and short, terminating in inflated nodus ramosus between et and est. Both chelal fingers with a row of acute teeth, spaced contiguously along the margin, slightly rounded proximally: fixed chelal finger with 24 teeth; movable chelal finger with 28 teeth (nearly as large as teeth on fixed chelal finger); without accessory teeth (Fig. 15G). Femur without long tactile setae. Movable chelal finger slightly curved in lateral view (Figs 14B, 15G). ***Opisthosoma***: typical, all setae long and acuminate; pleural membrane longitudinally striate, without setae. Tergites incompletely divided, tergal chaetotaxy I–XI: 10: 8: 5: 11: 13: 14: 15: 15: 17: 17(13 + 4T): 12(10 + 2T). Sternites X and XI undivided and other sternites divided, sternal chaetotaxy IV–XI: 5–5: 8–8: 8–8: 7–8: 7–7: 7–7: 18: 16; sternites X and XI each with two pairs of long tactile setae. Anus (tergite XII and sternite XII) without raised rim. Anterior genital operculum with five or seven setae on each side, posterior margin with eight setae. ***Legs*** (Figs 14G–I, 15E, F): generally typical, fairly smooth, slightly stout; junction between femora and patellae I and II oblique. Femoropatella of leg IV 2.80× longer than deep; tibia 3.86× longer than deep; with basal tactile setae on tarsal segment: tarsus 3.55× longer than deep (TS = 0.10); subterminal tarsal setae arcuate and acute. Arolium slightly shorter than claws, not divided; claws smooth.

**Adult females** (Figs 13B, 14E, 15I): mostly same as the male, but larger and paler. ***Chelicera***: galea distinct relatively short, with five distinct larger branchlets (Fig. 15C); serrula exterior with 25 lamellae, the basal one longest. ***Pedipalp***: stout, trochanter 1.67–1.78×, femur 2.42–2.52×, patella 2.00–2.25×, chela (with pedicel) 2.57–2.67×, chela (without pedicel) 2.37–2.42×, hand (with pedicel) 1.61–1.69×, hand (without pedicel) 1.41–1.44× longer than broad, movable chelal finger 0.58–0.60× (0.68×) longer than hand with pedicel (without pedicel) and 0.37× (0.40–0.41×) longer than chela with pedicel (without pedicel); palpal coxa with four apical setae, sub-terminal seta larger; all segments (except chelal fingers) with fine granulations. ***Opisthosoma***: tergites incompletely divided, tergal chaetotaxy I–XI: 8: 9: 10: 13: 16: 14: 15: 17: 15: 16(12 + 4T): 11(9 + 2T). Sternites divided, sternal chaetotaxy IV–XI: 7–8: 8–8: 8–8: 8–9: 8–8: 9–9: 16(12 + 4T): 14(12 + 2T). Genitalia simple, spermathecae provided with separated median cribriform plates; anterior genital operculum with five setae on each side, posterior margin with four or five setae on each side. ***Legs***: femoropatella of leg IV 2.81–2.93× longer than deep; tibia 3.28–3.41× longer than deep; with basal tactile setae on tarsal segment: tarsus 3.75–4.00× longer than deep (TS = 0.09–0.11).

**Dimensions** (length/breadth or, in the case of the legs, length/depth in mm). Male (females in parentheses): body length 2.73 (4.25–4.40). Carapace 0.85/0.71 (0.93–1.01/0.80–0.84). Pedipalp: trochanter 0.46/0.26 (0.45–0.48/0.27), femur 0.75/0.29 (0.75–0.78/0.31), patella 0.72/0.37 (0.72–0.81/0.36), chela (with pedicel) 1.28/0.47 (1.39/0.52–0.54), chela (without pedicel) 1.18 (1.26–1.28), hand (with pedicel) 0.78 (0.87–0.88), hand (without pedicel) 0.68 (0.75–0.76), movable finger length 0.50 (0.51–0.52). Leg I: trochanter 0.18/0.15 (0.17–0.20/0.14–0.17), femur 0.25/0.19 (0.24–0.26/0.20–0.21), patella 0.37/0.17 (0.41/0.18), tibia 0.39/0.12 (0.42–0.44/0.12), tarsus 0.38/0.09 (0.40–0.42/0.08–0.09). Leg IV: trochanter 0.29/0.16 (0.32–0.33/0.21), femoropatella 0.70/0.25 (0.76–0.79/0.27), tibia 0.54/0.15 (0.58–0.59/0.17–0.18), tarsus 0.39/0.11 (0.44–0.45/0.11–0.12).

##### Remarks.

*Catatemnustengchongensis* sp. nov. can be distinguished from *C.birmanicus* by the smaller body size (♂) (e.g., body length 2.73 mm vs 4.27 mm; palpal femur 2.59× vs 2.44× longer than broad, length 0.75 mm vs 1.09 mm; movable chelal finger length 0.50 mm vs 0.78 mm) and the trait of eyespots (with two distinct eyespots vs with two faint eyespots); from *C.concavus* by the trait of eyespots (with two distinct eyespots vs with two faint eyespots), the stouter pedipalps (♀) (e.g., palpal femur 2.42–2.52× vs 2.29× longer than broad, length 0.75–0.78 mm vs 0.90 mm) and the traits of the three first tergites (regularly convex from the one side to the other vs with a descending lateral portion, perpendicular on the dorsal part); from *C.monitor* by the trait of eyespots (with two distinct eyespots vs no eyespots visible) and the stouter pedipalps (e.g., palpal femur (♂) 2.59× vs 2.25×, (♀) 2.42–2.52× vs 2.27× longer than broad, length (♂) 0.75 mm vs 1.01 mm, (♀) 0.75–0.78 mm vs 0.95 mm); from *C.nicobarensis* by the trait of eyespots (with two distinct eyespots vs with two indistinct eyespots) and the traits of the palpal trochanter (anterior surface granular vs smooth or almost smooth); from *C.thorelli* by the trait of furrow on the carapace (distinctly curved vs almost straight) and the traits of pedipalps (surface of all segments except chelal fingers granular vs surface of palps smooth; palpal femur 2.59× vs 2.40× longer than broad, length 0.75 mm vs 1.15 mm; movable chelal finger teeth length 0.50 mm vs 0.70 mm) (Thorell 1889; Balzan 1891; With 1906; Beier 1932).

##### Distribution.

Known only from the type locality.

##### Etymology.

Named after the type locality, Tengchong (Yunnan).

#### ﻿ Catatemnus tibetanus sp. nov.

Taxon classificationAnimaliaPseudoscorpionesAtemnidae

7499EA7A-3D15-5778-8DC9-8A6A384841B2

https://zoobank.org/35D3757B-4668-4BA4-A512-7DB9EC52A87E

**Figure 16.**
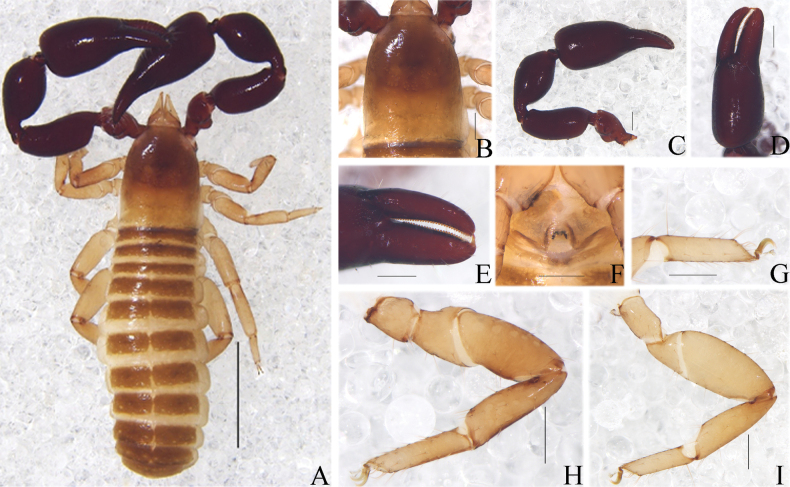
*Catatemnustibetanus* sp. nov., holotype male **A** habitus (dorsal view) **B** carapace (dorsal view) **C** left pedipalp (dorsal view) **D** left chela (lateral view) **E** left chela fingers (lateral view) **F** genital area (ventral view) **G** tarsus of left leg IV (lateral view) **H** left leg I (lateral view) **I** left leg IV (lateral view). Scale bars: 1.00 mm (**A**); 0.20 mm (**B–I**).

**Figure 17.**
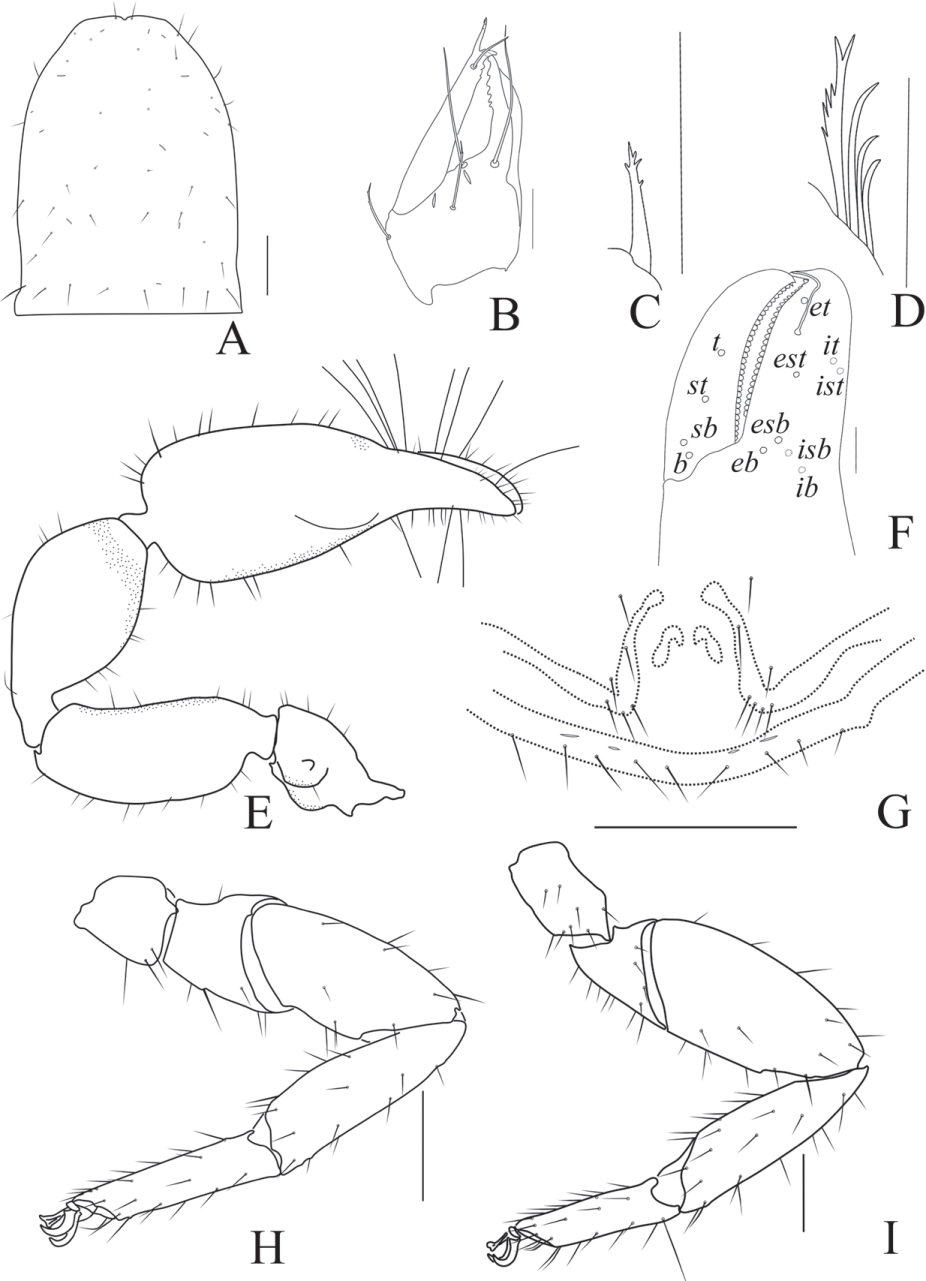
*Catatemnustibetanus* sp. nov., holotype male **A** carapace (dorsal view) **B** left chelicera (dorsal view) **C** galea **D** rallum **E** left pedipalp (dorsal view) **F** left chelal fingers (lateral view), with details of trichobothrial pattern **G** genital area (ventral view) **H** left leg I (lateral view) **I** left leg IV (lateral view). Scale bars: 0.20 mm (**A, E, G–I**); 0.10 mm (**B–D, F**). Abbreviations: **b** = basal; **sb** = sub-basal; **st** = sub-terminal; **t** = terminal; **ib** = interior basal; **isb** = interior sub-basal; **ist** = interior sub-terminal; **it** = interior terminal; **eb** = exterior basal; **esb** = exterior sub-basal; **est** = exterior sub-terminal; **et** = exterior terminal.

Figs 16 , 17 (西藏猫伪蝎)

##### Type material.

***Holotype***: China • ♂; Xizang Autonomous Region (Tibet), Motuo County, Beibeng Township, Jiangxin Village; 29°14.199'N, 95°10.740'E; 850 m a.s.l.; 09 Aug. 2015; Yejie Lin leg.; Ps.–MHBU–XZ15080901.

**Diagnosis** (♂). This new species is characterized by: two distinct developed eyespots, 1.32× longer than broad; palpal slightly stout, femur 2.44×, chela with pedicel 2.82×, chela without pedicel 2.62× longer than broad; distance between ist and isb farther to distance between est and esb; retrolateral surface of trochanter, prolateral surface of patella, femur and hand and base of chelal fingers granular.

##### Description.

**Adult male** (female unknown) (Figs 16, 17). ***Color***: anterior half of carapace and pedipalps reddish brown, posterior half of carapace yellowish brown; tergites brown. ***Carapace*** (Figs 16B, 17A): length distinctly longer than breadth (1.32×); anterior half darker than posterior half; surface smooth, but with a medial furrow; with two distinct eyespots situated near anterior margin of carapace; anterior margin with four setae, posterior margin with eight setae, 40 in total, each seta acicular and very slightly curved. ***Chelicera*** (Fig. 17B–D): much smaller than carapace length; surface smooth; four setae (sbs absent; es shorter than others) and two lyrifissures (exterior condylar lyrifissure and exterior lyrifissure) present on hand; movable finger with one galeal seta; bs dentate apically, is and ls long and acute. Fixed finger with five large retrorse teeth and two or three small apical teeth, movable finger with a long broadly rounded subapical lobe; galea present, short and apically with five branchlets (Fig. 17C). Serrula interior connected to fixed finger for entire length, proximally modified to form velum, serrula exterior with 27 lamellae, the basal one longest; lamina exterior present. Rallum comprising four blades, the basal two blades shorter than others, the distal one dentated anteriorly, remainder smooth (Fig. 17D). ***Pedipalp*** (Figs 16C–E, 17E, F): stout, trochanter 1.74×, femur 2.44×, patella 1.98×, chela with pedicel (without pedicel) 2.82× (2.62×), hand without pedicel 1.50× longer than broad; movable chelal finger 0.66× longer than hand with pedicel. Setae generally long and acuminate. Retrolateral surface of trochanter, prolateral surface of patella, femur, and hand granular; trochanter with two well-developed conical tubercles. Fixed chelal finger with eight trichobothria, movable chelal finger with four trichobothria: eb and esb situated at base of fixed finger on retrolateral face, esb slightly distal to eb; ib and isb situated at base of fixed finger on prolateral face, isb slightly distal to ib; est in the middle of fixed finger; et near sub-distal of fixed finger; est midway between et and esb; it distal to est and proximal to et; ist situated very slightly distal to est; it closer to ist than to fingertip; distance between est and esb shorter to that of ist and isb; b and sb situated at base of movable finger on retrolateral face; t in the middle of movable finger and at same level as est; sb closer to b than to st; st midway between t and sb (Fig. 17F). Venom apparatus only present in fixed chelal finger, venom ducts curved and short, terminating in inflated nodus ramosus between et and est. Both chelal fingers with a row of acute teeth, spaced contiguously along the margin, slightly rounded proximally: fixed chelal finger with 26 teeth; movable chelal finger with 32 teeth (nearly as large as teeth on fixed chelal finger); without accessory teeth (Fig. 17F). Sense spots absent; femur without long tactile setae. Movable chelal finger straight in lateral view (Figs 16E, 17F). ***Opisthosoma***: typical, surface irregular grid decoration, all setae long and acuminate. Pleural membrane longitudinally striate, without setae. Tergites I–III and XI undivided, tergal chaetotaxy I–XI: 8: 7: 9: 6–6: 6–6: 6–5: 7–6: 6–7: 7–5: 7–6 + (2T): 12 (4T). Sternites divided, sternal chaetotaxy IV–XI: 6–5: 9–9: 8–7: 8–8: 7–7: 8–7: 8–7 + (4T): 12 + (4T). Anus (tergite XII and sternite XII) without raised rim. Anterior genital operculum with six setae on each side, posterior margin with four marginal setae on each side. ***Legs*** (Figs 16G–I, 17H, I): typical, fairly smooth, slightly stout; junction between femora and patellae I and II oblique. Femoropatella of leg IV 2.67× longer than deep; tibia 3.53× longer than deep; with basal tactile setae on tarsal segment: tarsus 3.82× longer than deep (TS= 0.11); subterminal tarsal setae arcuate and acute. Arolium slightly shorter than claws, not divided; claws smooth.

**Dimensions** (length/breadth or, in the case of the legs, length/depth in mm). Male: body length 3.34. Carapace 0.99/0.75. Pedipalp: trochanter 0.47/0.27, femur 0.83/0.34, patella 0.81/0.41, chela (with pedicel) 1.41/0.50, chela (without pedicel) 1.31/0.50, hand (with pedicel) 0.85/0.50, hand (without pedicel) 0.75/0.50, movable chelal finger length 0.56. Leg I: trochanter 0.20/0.16, femur 0.26/0.20, patella 0.42/0.19, tibia 0.44/0.13, tarsus 0.38/0.09. Leg IV: femoropatella 0.80/0.30, tibia 0.60/0.17, tarsus 0.42/0.11.

##### Remarks.

*Catatemnustibetanus* sp. nov. can be distinguished from *C.birmanicus* by the smaller body size (♂) (e.g., body length 3.34 mm vs 4.27 mm; palpal femur length 0.83 mm vs 1.09 mm; movable chelal finger length 0.56 mm vs 0.78 mm) and the trait of eyespots (with two distinct eyespots vs with two faint eyespots); from *C.concavus* by the trait of eyespots (with two distinct eyespots vs with two faint eyespots) and the traits of pedipalps (the dorsal surfaces of femur and hand almost smooth vs with minute low granules everywhere except on fingers); from *C.monitor* by the trait of eyespots (with two distinct eyespots vs no eyespots visible) and the stouter pedipalps (♂) (e.g., palpal femur 2.44× vs 2.25× longer than broad, length 0.83 mm vs 1.01 mm); from *C.nicobarensis* by the trait of eyespots (with two distinct eyespots vs with two indistinct eyespots) and the traits of the palpal trochanter (anterior surface granular vs smooth or almost smooth); from *C.thorelli* by the trait of furrow on the carapace (distinctly curved vs almost straight) and the stouter pedipalps (e.g., palpal femur 2.44× vs 2.82× longer than broad, length 0.83 mm vs 0.96 mm; movable chelal finger teeth length 0.56 mm vs 0.70 mm) (Thorell 1889; Balzan 1891; With 1906; Beier 1932).

##### Distribution.

Known only from the type locality.

##### Etymology.

Named after the type locality, Tibet (China).

### ﻿Key to the species of *Catatemnus* from China

**Table:** 

1	Carapace with two well-developed eyespots	**2**
–	Carapace with two faint eyespots	***C.huae* sp. nov.**
2	The distal two rallum blades dentated anteriorly	***C.ramus* sp. nov.**
–	The distal one rallum blade dentated anteriorly	**3**
3	Distance between est and esb shorter to that of ist and isb	***C.tibetanus* sp. nov.**
–	Distance between est and esb equal or farther to that of ist and isb	**4**
4	Serrula exterior with 27 or 29 lamellae and length of chelal hand (with pedicel) > 1.00 mm	***C.laminosus* sp. nov.**
–	Serrula exterior with 24 or 25 lamellae and length of chelal hand (with pedicel) < 1.00 mm	**5**
5	Movable chelal finger with 28 teeth; chelal hand with abundant granulation	***C.tengchongensis* sp. nov.**
–	Movable chelal finger with 32 teeth; chelal hand with granulation only laterally	***C.scaber* sp. nov.**

## Supplementary Material

XML Treatment for Catatemnus

XML Treatment for Catatemnus huae

XML Treatment for Catatemnus laminosus

XML Treatment for Catatemnus ramus

XML Treatment for Catatemnus scaber

XML Treatment for Catatemnus tengchongensis

XML Treatment for Catatemnus tibetanus

## ﻿Additional information

### Author ORCIDs

Yanmeng Hou https://orcid.org/0000-0003-0059-3419

Lingchen Zhao https://orcid.org/0000-0001-8477-836X

Feng Zhang https://orcid.org/0000-0002-3347-1031

### Data availability

All of the data that support the findings of this study are available in the main text.
